# Bilirubin Sensing Using Organic Electrochemical Transistors: Role of Gate Materials and Operational Parameters

**DOI:** 10.1002/adhm.202502481

**Published:** 2025-08-07

**Authors:** Yunjia Song, Sihui Xu, Onur Parlak

**Affiliations:** ^1^ Department of Medicine Solna Division of Dermatology and Venereology Karolinska Institutet Stockholm 17177 Sweden; ^2^ Centre for Molecular Medicine Karolinska University Hospital Stockholm 17176 Sweden; ^3^ Center for the Advancement of Integrated Medical and Engineering Sciences Karolinska Institutet and KTH Royal Institute of Technology Stockholm 10044 Sweden

**Keywords:** bilirubin sensing, biosensor, electrochemistry, hyperbilirubinemia, OECT

## Abstract

Bilirubin, a critical yellow‐orange bile pigment and heme degradation product, serves as a key biomarker for neonatal jaundice and liver dysfunction, with elevated concentrations posing significant neurotoxicity risks particularly in neonates. However, timely detection remains challenging due to limitations in current point‐of‐care technologies. This study reveals that organic electrochemical transistors that include PEDOT:PSS as the channel material exhibit inherent sensitivity to free bilirubin ‐ but only when paired with polarizable gate electrodes (Au, Pt, glassy carbon). Intriguingly, this response is abolished with non‐polarizable Ag/AgCl gates, highlighting the pivotal role of electrode polarizability in bilirubin detection. Furthermore, the drain‐source current changing direction is modulated by operational parameters, suggesting complex interfacial dynamics between bilirubin and channel material. Through systematic investigation, we demonstrate that this sensitivity persists for human serum albumin‐bound bilirubin, a clinically relevant complex, and elucidate the redox mechanisms underlying signal transduction via cyclic voltammetry. Our work not only decouples the influence of gate materials and measurement conditions on device performance but also establishes a foundational framework for designing high‐precision bilirubin sensors, paving the way for transformative diagnostic devices that address critical gaps in neonatal care through miniaturized, low‐power platforms capable of real‐time bilirubin monitoring in both clinical and resource‐limited settings.

## Graphical abstract

This study explores PEDOT:PSS‐based OECTs as sensitive bilirubin sensors, revealing that polarizable gate electrodes (Au, Pt, glassy carbon) enable detection while non‐polarizable Ag/AgCl gates suppress response. Operational parameters modulate current direction, suggesting complex interfacial dynamics. The sensors detect clinically relevant HSA‐bound bilirubin, with redox mechanisms elucidated via cyclic voltammetry, advancing low‐power, real‐time neonatal monitoring solutions.

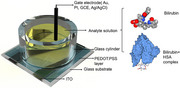

## 1 Introduction

**Scheme 1:**
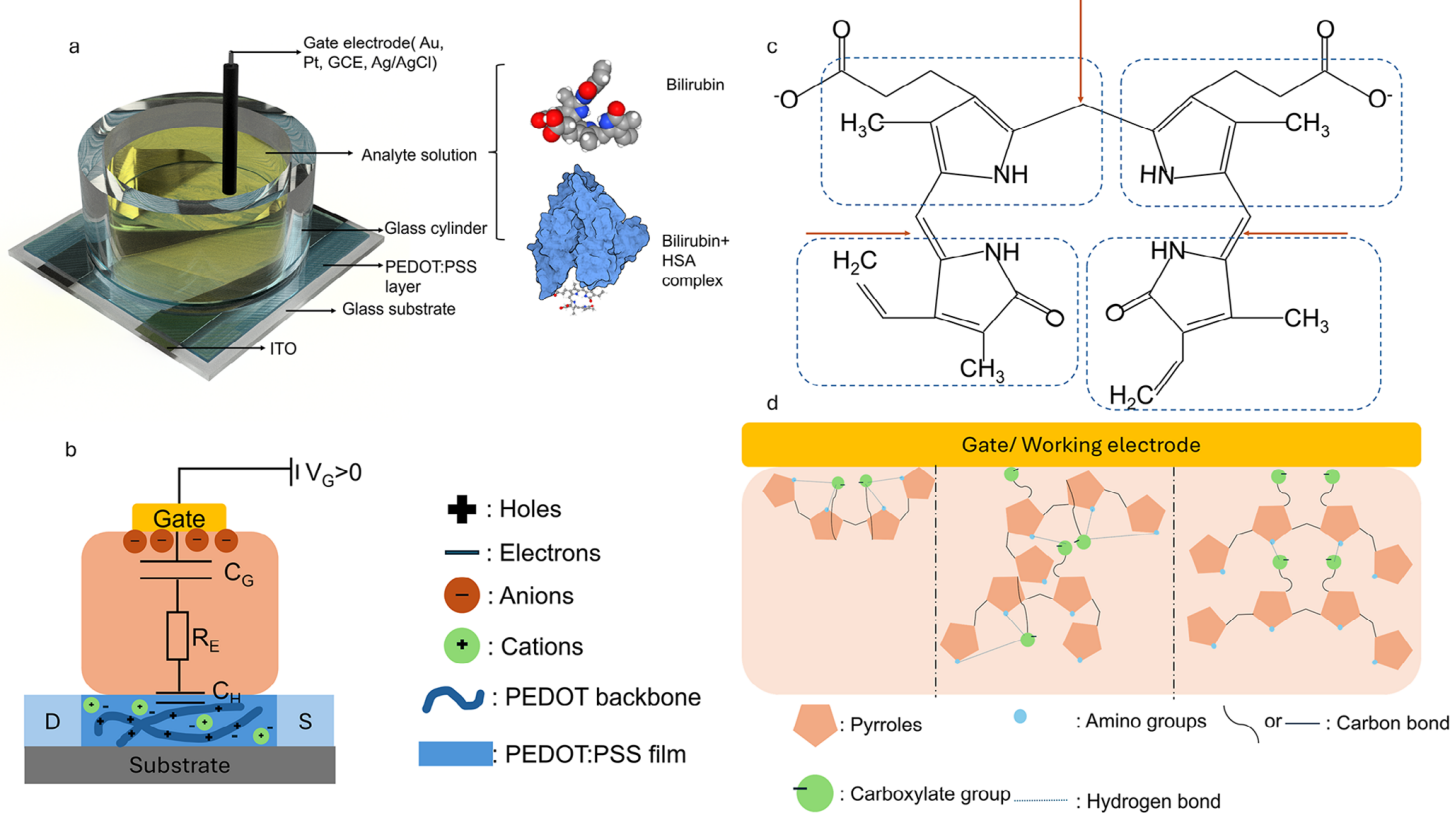
a) Schematic illustration of the OECT setup and analyte used in this work. b) Schematics of electrical components in OECT measurements. c) Molecular structure of bilirubin in 0.1 M KCl (pH 7.7) solution. d) Possible mechanisms of redox reaction close to the gate/working electrode.

Neonatal jaundice is a prevalent physiological or pathological phenomenon in newborns, with documented studies tracing back to the 19th century.^[^ ^1^, ^2^, ^3^ ^]^ Physiological neonatal jaundice is typically benign and self‐resolving, whereas pathological jaundice may progress to severe neonatal hyperbilirubinemia (SNH), potentially culminating in kernicterus, hypotonia, and irreversible neurological sequelae.^[^ ^3^, ^4^, ^5^ ^]^ The clinical presentation of jaundice is characterized by a yellowish discoloration of the skin, conjunctiva, and other tissues, resulting from the accumulation of total serum bilirubin (TSB).^[^ ^6^, ^7^ ^]^ Bilirubin (BR), a yellow to orange tetrapyrrole bile pigment, is a catabolic byproduct of heme degradation, predominantly derived from hemoglobin. Its formation involves a two‐step enzymatic process: initial oxidative cleavage of heme by heme oxygenase (HMOX) yields biliverdin, carbon monoxide, and ferrous iron, followed by subsequent reduction of biliverdin by biliverdin reductase to generate unconjugated bilirubin (**Scheme** **1**).^[^ ^8^, ^9^, ^10^, ^11^, ^12^ ^]^ The propensity of unconjugated bilirubin to form intramolecular hydrogen bonds restricts its aqueous solubility under physiological conditions, necessitating non‐covalent binding to serum albumin for hepatic transport. Subsequent glucuronidation by hepatic uridine diphosphate‐glucuronosyltransferase (UGT) enzymes yields water‐soluble conjugated bilirubin, which undergoes biliary excretion.^[^ ^13^, ^14^, ^15^ ^]^

In healthy adults and children, TSB concentrations range from 0.2 to 1.2 mg dL^−1^ (3.42–20.52 µM) and remain below 1.5 mg dL^−1^ (26 µM), respectively. However, neonates exhibit higher bilirubin levels during the transitional period, with clinically apparent jaundice manifesting at TSB concentrations exceeding 5 mg dL^−1^ (86 µM). Therapeutic intervention becomes imperative when TSB surpasses 15 mg dL^−1^ to mitigate the risk of bilirubin‐induced neurological dysfunction.^[^ ^16^, ^17^ ^]^

Quantification of bilirubin in biological matrices has been achieved through diverse analytical methodologies, including spectroscopic,^[^ ^18^ ^]^ chromatographic,^[^ ^19^ ^]^ capillary electrophoretic,^[^ ^20^ ^]^ and electrochemical^[^ ^21^ ^]^ techniques. The diazo reaction, which exploits the formation of azobilirubin chromophores for spectrophotometric detection, remains the conventional reference method.^[^ ^22^, ^23^, ^24^ ^]^ Nevertheless, these approaches are constrained by their reliance on sophisticated instrumentation, specialized technical expertise, and protracted analysis times, thereby underscoring the need for point‐of‐care (POC) alternatives.^[^ ^25^ ^]^ While optical and chemical POC detection strategies have been extensively investigated, electrochemical POC platforms remain comparatively underexplored.^[^ ^26^, ^27^, ^28^, ^29^ ^]^ Electrochemical biosensors offer distinct advantages, including rapid response kinetics and compatibility with miniaturized readout systems.^[^ ^30^ ^]^ Predominant electrochemical configurations employ screen‐printed electrodes (SPEs) functionalized with either enzymatic (e.g., bilirubin oxidase (BOx) immobilized on polymeric^[^ ^31^ ^]^ or nanostructured substrates^[^ ^32^ ^]^) or non‐enzymatic recognition elements (e.g., graphene‐polymer composites,^[^ ^33^ ^]^ molecularly imprinted polymers,^[^ ^34^ ^]^ metallic nanorods,^[^ ^35^ ^]^ or carbon nanotube‐metal nanoparticle hybrids^[^ ^36^ ^]^). These platforms typically employ voltammetric (cyclic voltammetry (CV), linear sweep voltammetry (LSV), amperometric), or electrochemical impedance spectroscopy (EIS) detection modalities.^[^ ^31^, ^32^, ^33^, ^34^, ^35^, ^36^ ^]^

Besides conventional electrochemical measurements, organic electrochemical transistors (OECTs) have emerged as a promising biosensing paradigm due to their low operational voltages, exceptional transconductance, and superior sensitivity to ionic‐electronic coupling phenomena. The OECT architecture comprises three terminals (gate, drain, source), with the channel region consisting of organic mixed ionic‐electronic conductors (OMIECs), most notably the conjugated polymer poly(3,4‐ethylenedioxythiophene): polystyrene sulfonate (PEDOT:PSS). It has also been widely used in wearable electronics because of its relatively high robustness under external mechanical forces.^[^ ^36^, ^37^, ^38^, ^39^, ^40^, ^41^, ^42^, ^43^, ^44^, ^45^, ^46^ ^]^ Despite their potential, OECT‐based bilirubin detection remains scarcely reported. Cai et al. demonstrated a photoelectrochemical transistor employing metal‐organic framework (MOF)‐modified TiO_2_ nanorod arrays as the gate electrode, wherein bilirubin sensitivity was achieved through photoinduced electron transfer.^[^ ^47^ ^]^ However, the mechanism of purely electrically driven bilirubin detection in OECT configurations remains almost uninvestigated. Furthermore, most OECT‐based metabolite sensors utilize polarizable gate electrodes (e.g., Pt, Au), leaving the influence of gate material selection on metabolite‐channel interactions largely unexplored.^[^ ^48^, ^49^ ^]^

In this study, we systematically evaluate the modulation of drain‐source current in OECTs in response to different concentrations of free bilirubin and human serum albumin‐bilirubin (HSA‐BR) complexes. A standardized OECT architecture was constructed with indium tin oxide (ITO) drain/source electrodes and a PEDOT:PSS channel (Scheme 1a). To investigate the role of gate electrode polarizability, four distinct gate materials—Au, Pt, glassy carbon (GCE), and Ag/AgCl—were comparatively assessed. Comprehensive characterization was performed across a range of drain‐source (V_D_) and gate (V_G_) voltages to delineate the operational parameters influencing bilirubin‐PEDOT:PSS interactions. This foundational study provides critical insights into the mechanisms of OECT‐based bilirubin detection, thereby facilitating the rational development of robust, clinically translatable biosensing platforms.

## 2 Results and Discussion

### 2.1 OECT‐Based Bilirubin Sensing: Gate Electrode and Voltage Effects

The investigation of free bilirubin sensing using OECTs is a crucial step toward developing advanced biosensors for neonatal jaundice monitoring. As stated in the previous section, OECTs offer high sensitivity for constructing bilirubin sensors. Our experimental approach began with comprehensive measurements using free bilirubin solutions to establish fundamental performance characteristics.

**Figure 1:**
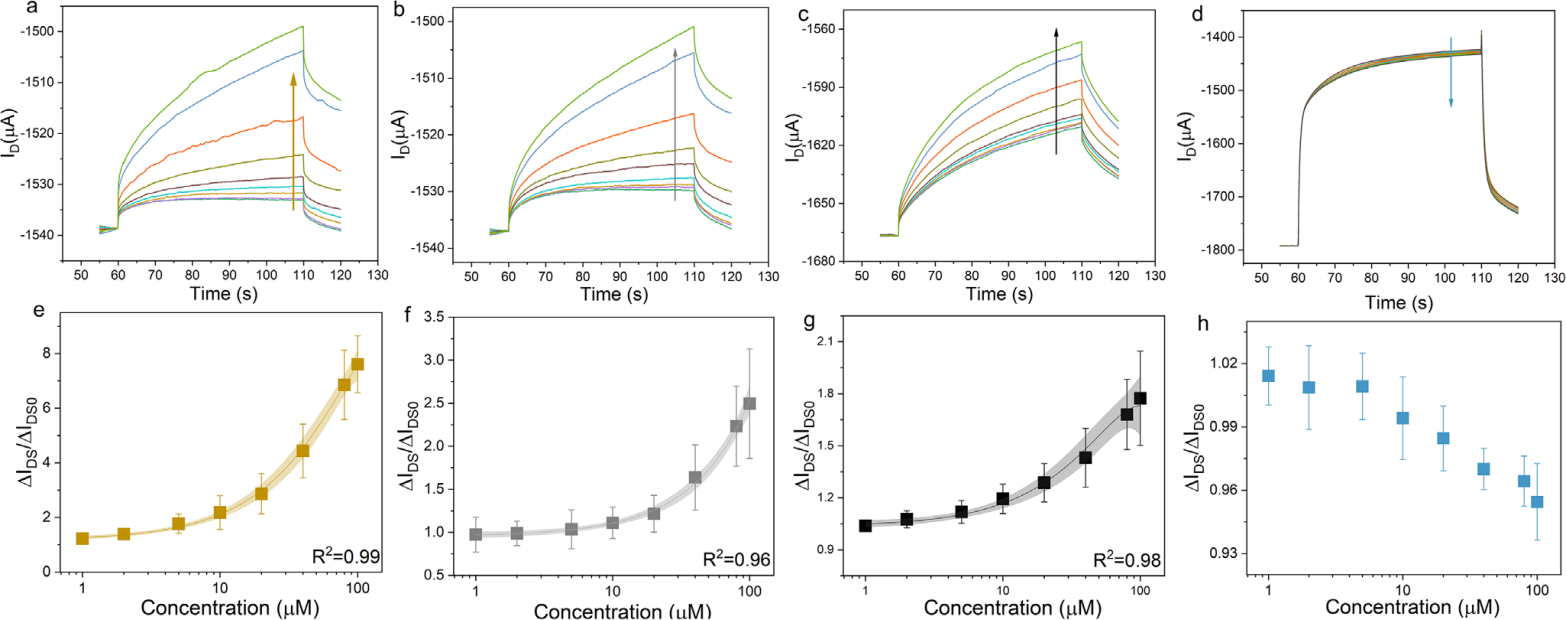
Drain‐source current change vs time with a) Au b) Pt c) GC d) Ag/AgCl gate electrodes. The arrows indicate the direction of current change vs different free bilirubin solution concentrations (blank‐1µM‐2µM‐5µM‐10µM‐20µM‐40µM‐80µM‐100µM). Drain‐source current ratio changes vs different free bilirubin solution concentrations with e) Au f) Pt g) GC h) Ag/AgCl gate electrode. All measurements were done under V_D_=−0.3 V V_G_=0.3 V with free bilirubin solution. The x‐axis is shown in log form for e‐h.

The initial measurements were intentionally conducted under relatively small voltage conditions (V_D_ = ‐0.3 V and V_G_ = 0.3 V) to systematically compare how different gate electrode materials affect the interaction between free bilirubin molecules and the PEDOT:PSS channel in OECTs. We selected four distinct electrode types for this comparative analysis: gold (Au), platinum (Pt), glassy carbon electrode (GCE), and Ag/AgCl electrodes. The complete set of results obtained from these different gate electrodes is presented in **Figure** **1**.

Figure 1a‐d clearly demonstrates the changes in drain‐source current that occur in response to increasing concentrations of free bilirubin solutions. It is important to clarify that our “free bilirubin solution” refers to bilirubin dissolved in 0.1M KCl aqueous solution with the assistance of concentrated NaOH solution. This preparation method is necessary because bilirubin exhibits extremely limited solubility in water under physiological pH conditions.^[^ ^8^, ^9^, ^10^, ^11^, ^12^ ^]^ The addition of basic solutions serves to disrupt the internal hydrogen bonds of bilirubin molecules, thereby significantly enhancing their solubility in the aqueous medium. In this carefully prepared system, the solution contains only bilirubin molecules, water molecules, and several ionic species (K^+^, Cl^−^, Na^+^, H^+^, and OH^−^). We maintained the pH of the free bilirubin solution at 7.7 ± 0.05 throughout all experiments, as higher pH values could potentially affect the electrical properties of PEDOT:PSS.^[^ ^50^ ^]^

The experimental results reveal striking differences in device behavior depending on the gate electrode material. For the polarizable gate electrodes (Au, Pt, and GCE), we observed that after applying the gate voltage, the magnitude of the drain‐source current change increased proportionally with bilirubin concentration. In marked contrast, when using the Ag/AgCl gate electrode, the current change showed only a slight decreasing tendency in absolute value, with the scale of these changes being essentially negligible compared to the polarizable electrodes.

To quantify these observations, we performed detailed analysis using current ratio changes, as shown in Figure 1e‐h. In our notation, ΔI_DS_ represents the current change measured in different concentrations of free bilirubin solution after gate potential application, while ΔI_DS0_ corresponds to the current change measured in pure 0.1M KCl solution (blank measurement) under identical gate potential conditions. The sensitivity to free bilirubin is therefore properly expressed by the current ratio of ΔI_DS_/ΔI_DS0_. The calibration curves derived from this analysis (each generated from at least five independent samples) demonstrate that among all polarizable gate electrodes, the Au electrode provides maximum sensitivity, achieving an average current ratio of 8. The GCE showed minimum sensitivity among polarizable electrodes, with an average current ratio reaching only 1.75, while the Pt electrode exhibited intermediate sensitivity with a maximum average current ratio change of 2.5. The Ag/AgCl electrode, as noted previously, produced only minimal changes in the current ratio.

To ensure that the observed current ratio changes truly resulted from bilirubin‐PEDOT:PSS interactions rather than potential ionic accumulation effects in the channel region, we conducted an extensive series of control experiments. These control measurements followed exactly the same protocol but used solutions containing only the ionic components without any bilirubin. The results of these critical control experiments are presented in Figures S1 and S2 (Supporting Information). The control data demonstrate that for the three polarizable electrodes, any changes in current ratio either showed no consistent trend or exhibited drift magnitudes negligible compared to those observed in the bilirubin‐containing experiments. However, for the Ag/AgCl electrode, the current ratio changes observed in control experiments were similar in scale to those seen in the bilirubin‐containing measurements. This comparison conclusively shows that the current changes corresponding to bilirubin concentration are fundamentally dependent on the type of gate electrode used during measurement.

We identified several potential origins for these observed variations: (1) differences in gating effects between electrode types, (2) varying interactions between the electrode materials and bilirubin molecules, and (3) possible bilirubin‐induced de‐doping effects on PEDOT:PSS. To further investigate how operational parameters affect device performance, we conducted an additional series of experiments examining the influence of higher applied voltages. These measurements used relatively large voltage magnitudes (V_D_ = ‐0.6 V and V_G_ = 0.8 V for Au, Pt, and GCE; V_D_ = ‐0.6 V and V_G_ = 0.4 V for Ag/AgCl electrode). The specific voltage values were carefully selected based on two considerations: first, these voltages correspond to the maximum transconductance region for OECTs with Au gate electrodes; second, we observed that PEDOT:PSS films undergo visible color changes when V_G_ exceeds 0.4 V with Ag/AgCl gate electrodes, necessitating the use of lower gate voltage for this electrode type. The complete set of transfer curves and transconductance calculations under different V_D_ values is provided in Figures S10 and S11 (Supporting Information) for reference.

**Figure 2:**
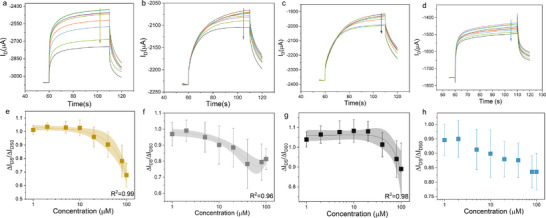
Drain‐source current change vs time with a) Au b) Pt c) GC d) Ag/AgCl gate electrodes. The arrows indicate the direction of current change vs different bilirubin concentrations (blank‐1µM‐2µM‐5µM‐10µM‐20µM‐40µM‐80µM‐100µM). Drain‐source current ratio changes vs different bilirubin concentration with e) Au f) Pt g) GC h) Ag/AgCl gate electrodes. Measurements with Au, Pt, and GC electrodes were done under V_D_=‐0.6V, V_G_=0.8V; with Ag/AgCl electrode were done under V_D_=‐0.6V, V_G_=0.4V. The measurements were done with free bilirubin solution. The x‐axis is shown in log form for e‐h.

The results obtained under these higher voltage conditions are presented in **Figure** **2**. Figure 2a‐d shows the time‐dependent drain‐source current changes as bilirubin concentration gradually increased. Surprisingly, under these elevated voltage conditions, we observed an opposite trend compared to low‐voltage measurements: instead of increasing in absolute value, the drain‐source current magnitude decreased with increasing bilirubin concentration. We plotted calibration curves using the same ΔI_DS_/ΔI_DS0_ ratio method as described earlier, with each curve again representing data from at least five independent samples. These curves reveal that under high V_D_ (absolute value) and V_G_ conditions, all four electrodes showed decreasing current ratios with increasing bilirubin concentration, while maintaining the same relative sensitivity ranking (Au > Pt > GCE > Ag/AgCl). Specifically, the Au gate electrode demonstrated the strongest response with a minimum current ratio reaching 0.6, while results from Pt and GCE electrodes showed minimum current ratios of 0.8 and 0.85, respectively.

We performed corresponding control experiments under these high voltage conditions, repeating the identical measurement process using a blank solution (without free bilirubin). These control results are presented in Figure S4 (Supporting Information). For the three polarizable electrodes, the control experiments either showed no significant drift trend or exhibited drift in the opposite direction compared to the bilirubin‐containing experiments. The Ag/AgCl electrode, however, displayed similar current ratio changes in both control and bilirubin‐containing experiments, strongly suggesting that its observed decreases in current ratio likely resulted from measurement drift rather than specific bilirubin interactions.

To further confirm that the sensitivity of the free bilirubin molecules does not come from the morphological change of the PEDOT:PSS layer, and also to prove the stability of the channel region under electrical measurements, the SEM images were taken before and after measurements under different gate voltages. The SEM images are shown in Figure S14 (Supporting Information), which indicate that the channel region maintains almost the same morphology before and after electrical measurement, and prove the robustness of the PEDOT:PSS layer under the application of potential.

These comprehensive experiments reveal two key phenomena: first, OECTs show clear sensitivity to bilirubin under both low and high voltage conditions; second, the direction of current ratio change completely reverses between these voltage regimes. This unexpected finding highlights the need for a deeper understanding of the underlying mechanisms. We hypothesize several possible explanations: 1) voltage‐dependent modulation may alter bilirubin's molecular structure differently at various potentials, 2) bilirubin may undergo different redox reactions that change its ability to dope or de‐dope PEDOT:PSS depending on applied voltages, or 3) the combination of gate potential and electrode materials may create distinct interfacial conditions that affect charge transfer processes differently. These complex interactions between bilirubin and OECT components under varying electrical conditions present both challenges and opportunities for optimizing biosensor design.

### 2.2 Validating Bilirubin Redox Mechanisms by Cyclic Voltammetry

To elucidate the electrochemical behavior of bilirubin during OECT operation and validate whether redox reactions contribute to the observed current modulations, we performed cyclic voltammetry (CV) measurements under identical experimental conditions. This analysis provides direct evidence of bilirubin's redox activity at different electrode materials while excluding potential artifacts from ionic interference, thereby clarifying the origin of the OECT signal generation.

In these measurements, the Au, Pt, and glassy carbon electrodes functioned as working electrodes, complemented by an Ag/AgCl reference electrode and Pt wire counter electrode in the electrolyte. We used the identical free bilirubin solution preparation method as in the OECT experiments, maintaining consistency without adding any mediators to the system.

**Figure 3:**
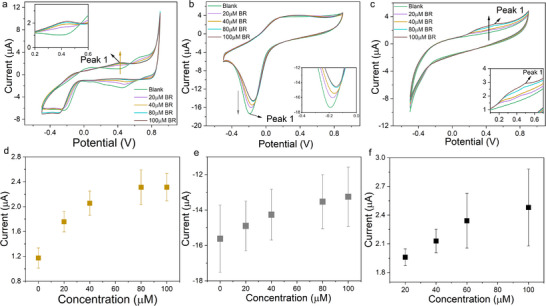
CV curves measured with different free bilirubin concentrations with a) Au b) Pt c) GC working electrodes. Peak current change vs free bilirubin concentration with d) Au e) Pt f) glassy carbon working electrode.

The CV results and derived calibration curves are presented in **Figure** **3**. Analysis of Figure 3a‐c reveals distinct redox activity for each electrode material within specific potential ranges. The Au electrode shows redox activity between 0.4 V and 0.7 V (Figure 3a), while the Pt electrode exhibits a clear redox peak at −0.18 V (Figure 3b). The GCE demonstrates redox behavior spanning 0.2 to 0.6 V (Figure 3c). We used the marked peak currents from these figures to generate calibration curves quantifying the relationship between current response and free bilirubin concentration.

Figure 3d‐f demonstrates that all three electrodes show increasing peak currents with rising bilirubin concentrations. To account for potential ionic interference, we performed control experiments following identical procedures but with bilirubin‐free solutions (results shown in Figure S5, Supporting Information). These control measurements produced nearly overlapping CV curves during repeated scans for all three polarizable electrodes. However, calibration data analysis revealed important distinctions: for Au and Pt electrodes, control experiments showed current drifts opposite to the bilirubin concentration‐dependent responses, while GCE control measurements exhibited minor current changes in the same direction but with much smaller magnitude.

We observed that even in control experiments, Au and Pt electrodes displayed small fluctuations, likely caused by ionic effects such as Cl‐ or OH‐ adsorption/etching processes.^[^ ^51^, ^52^ ^]^ For the Au electrode, when the concentration of free bilirubin solution was increased, new peaks arose, and the original peaks measured in KCl solution gradually disappeared, which indicates that there is a redox reaction close to the Au working electrode, and charge transfer between the Au electrode and bilirubin molecules becomes the major reaction. The new peak appears ≈0.4V. For the Pt electrode, it has a small peak created between ≈0.4V, and the peak intensity measured in KCl solution gradually decreased, which indicates that the redox reaction can also happen between the working electrode and bilirubin molecules. For the glassy carbon electrode, the peaks of the CV curves only show up after the addition of bilirubin molecules. Unlike metal electrodes, there may be no electron transfer between chloride ions can the carbon surface, but after the addition of bilirubin, the redox reaction happens and new peaks between 0.2 and 0.5 V show up. However, how strong the reaction is and to how much extent the interaction between bilirubin and the working electrode can become dominant may vary because of the material property. For the two metal electrodes, the charge transfer from the redox reaction is also influenced by the chloride ions in the electrolyte, and the influence might be different, so although they both have a new peak ≈0.4 V, the potential can be slightly different. As for the glassy carbon electrode, the chloride ions do not have an obvious influence on the process of charge transfer, so almost all the charge transfer between bilirubin and working electrodes can be measured, which explains why the peaks of CV curves with GCE electrodes show different positions. This theory also explains the reason that they have various sensitivities as gate electrodes in the OECT platform.

The CV results provide conclusive evidence that free bilirubin undergoes redox reactions when subjected to external voltages in this system. Considering bilirubin's known isomerization properties, these redox peaks may correspond to structural transitions between different bilirubin isomers under applied potentials.^[^ ^53^ ^]^ This comprehensive electrochemical characterization complements our OECT findings by providing direct evidence of bilirubin's redox activity under measurement conditions, while systematically ruling out alternative explanations through controlled experiments. The voltage‐dependent redox behavior observed here may help explain the contrasting OECT responses seen under different operational parameters.

### 2.3 Differential OECT Response to Protein‐Bound Versus Free Bilirubin

While our free bilirubin experiments demonstrated clear OECT responses, clinical relevance demands understanding how albumin‐bound bilirubin, the predominant form in human fluids‐interacts with the device. This investigation is crucial because human serum albumin (HSA) binds bilirubin with high affinity, potentially altering its electrochemical behavior and device response compared to free bilirubin.^[^ ^54^, ^55^ ^]^

**Figure 4:**
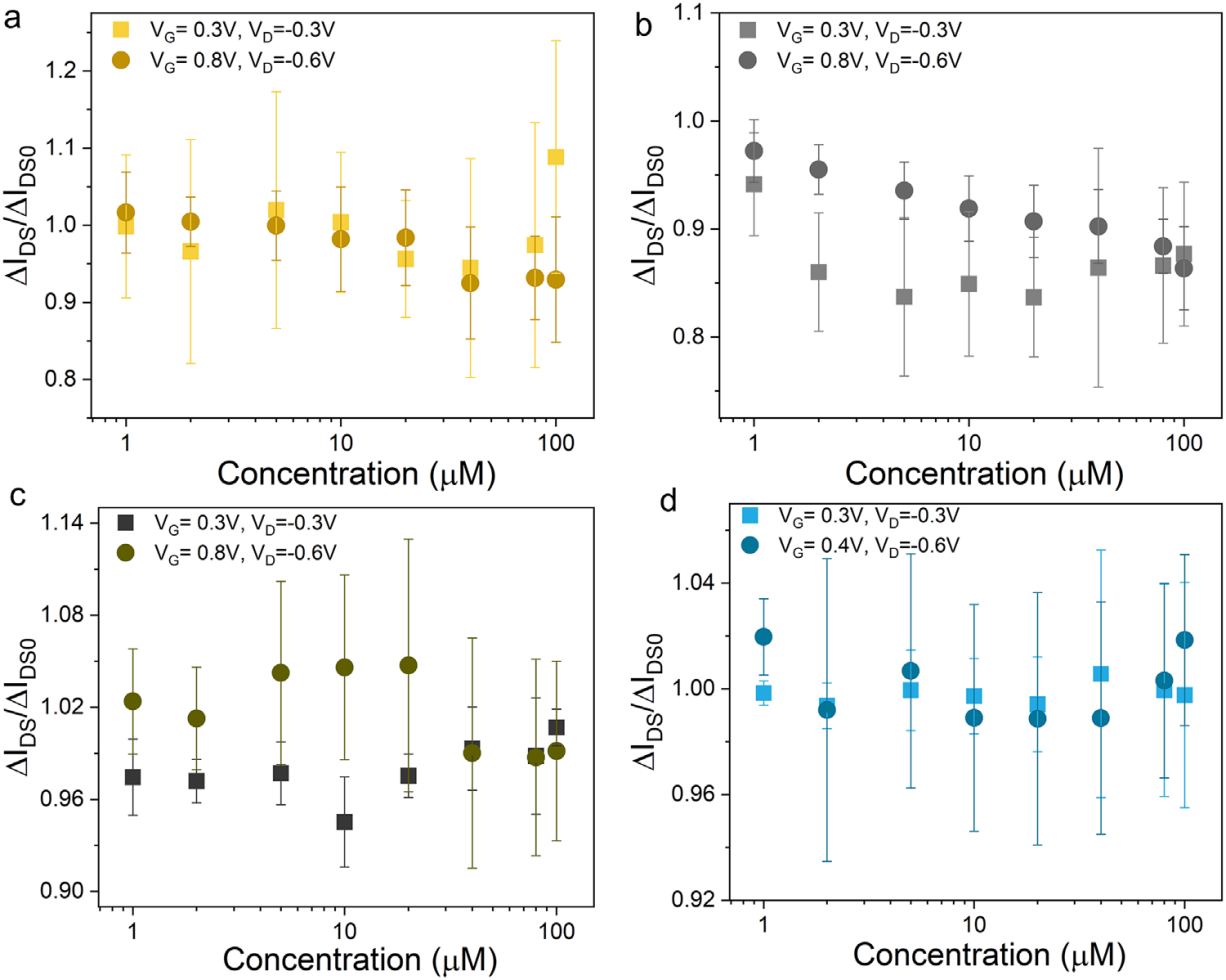
Drain‐source current ratio changes vs different HSA‐BR complex concentrations with a) Au b) Pt c) GC d) Ag/AgCl gate electrode. Measurements with Au, Pt, and GC electrodes were done under V_D_=−0.3 V, V_G_=0.3 V and V_D_=−0.6 V, V_G_=0.8 V; measurements with Ag/AgCl electrode were done under V_D_=−0.3 V, V_G_=0.3 V and V_D_=−0.6 V, V_G_=0.4 V. The x‐axis is shown in log form a‐d.

To evaluate whether the HSA‐bilirubin (HSA‐BR) complex modulates OECT current similarly to free bilirubin, we prepared solutions by dissolving HSA in 0.1M KCl. The 100 µM HSA‐BR complex solution contained equimolar concentrations of both HSA and bilirubin (100 µM each), with full experimental details provided in Section 3. **Figure** **4** presents the OECT results for HSA‐BR complex detection, following the identical measurement protocol used for free bilirubin. Each subfigure displays current ratio changes (ΔI_DS_/ΔI_DS0_) versus HSA‐BR concentration, with data acquired under both low (V_D_ = ‐0.3 V, V_G_ = 0.3 V) and high (V_D_ = −0.6 V, V_G_ = 0.8 V for Au/Pt/GCE; V_D_ = −0.6 V, V_G_ = 0.4 V for Ag/AgCl) voltage conditions. The complete time‐dependent current traces are shown in Figures S6 and S7 (Supporting Information).

The results reveal fundamentally different behavior compared to free bilirubin: across all four electrodes and both voltage regimes, current ratios fluctuated inconsistently between 0.8 and 1.1 without concentration‐dependent trends. The sole exception was Pt gate electrodes under high voltage, which showed a slight decreasing trend (current ratio ≈0.8 at the highest concentration). However, control experiments with HSA‐only solutions (Figure S8, Supporting Information) demonstrated identical magnitude and direction of current ratio changes for Pt electrodes, confirming this drift likely stems from measurement artifacts rather than HSA‐BR detection.

These collective findings demonstrate that HSA‐BR complex formation profoundly alters bilirubin's interaction with PEDOT:PSS compared to free bilirubin. We attribute this to HSA‐induced modifications in bilirubin's charge distribution and redox accessibility when bound, effectively suppressing the current modulation effects observed with free bilirubin. This has critical implications for clinical OECT biosensor development, as it necessitates distinct detection strategies for bound versus unbound bilirubin fractions.

### 2.4 HSA Complexation Alters Bilirubin's Redox Activity and Optical Properties

**Figure 5:**
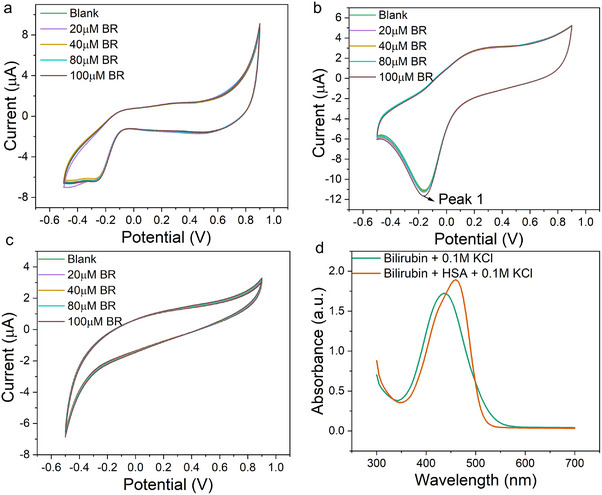
CV curves measured with different concentrations of HSA‐bilirubin complex with a) Au b) Pt c) GC electrodes. d) UV–vis spectrum of 100 µM free bilirubin solution and 100 µM HSA‐BR complex solution.

To investigate why HSA complexation dramatically alters bilirubin's electrochemical activity in OECTs, we conducted complementary CV and UV–vis spectroscopy studies. These investigations reveal how protein binding modifies bilirubin's structure and redox properties‐critical for developing clinically viable biosensors that must detect both free and protein‐bound fractions. The results are presented in **Figure** **5**. The CV measurements show nearly overlapping curves across increasing HSA‐BR concentrations (20‐100 µM), with no new redox peaks emerging compared to free bilirubin solutions. Quantitative analysis of current changes versus concentration (Figure S9, Supporting Information) confirms this observation‐while minor current fluctuations occur within a narrow range (variation <15%), no concentration‐dependent trends emerge. These CV results demonstrate two key findings: 1) the HSA‐BR complex exhibits no detectable redox activity under applied potential (‐0.5‐0.9 V), and 2) the solution contains negligible free bilirubin, as any unbound fraction would generate redox peaks matching our free bilirubin controls.

This electrochemical stability suggests the HSA‐BR complex either adopts a more rigid structure or experiences charge redistribution that: a) prevents electron transfer to/from electrodes, and b) minimizes field‐effect interactions with PEDOT:PSS. To test for structural modifications, we compared UV–vis spectra of free bilirubin versus HSA‐BR solutions (Figure 5d). The free bilirubin solution shows a characteristic absorption peak at 450 nm, while the HSA‐BR complex exhibits a redshifted peak at 480 nm with a shoulder at 430 nm. Control measurements of pure 0.1 M KCl solution and 100µM HSA solutions (Figure S12, Supporting Information) confirm these features originate from bilirubin: 0.1M KCl solution shows no peaks, while HSA exhibits only a weak 300 nm peak. These spectral shifts align with literature reports and indicate substantial structural reorganization of bilirubin upon HSA binding.^[^ ^53^, ^56^, ^57^ ^]^

The observed 30 nm redshift and peak broadening likely reflect multiple phenomena: 1) hydrogen bonding between bilirubin's carboxyl/pyrrole groups and HSA amino acid residues (e.g., Lys, Arg), and 2) encapsulation of bilirubin within HSA's hydrophobic binding pockets. Both mechanisms would alter bilirubin's conjugate π‐system and dipole moment, explaining both the spectroscopic changes and loss of electrochemical activity. This comprehensive characterization confirms that HSA complexation fundamentally modifies bilirubin's molecular and electronic structure, necessitating alternative detection strategies for clinical OECT biosensors targeting protein‐bound bilirubin.

## 3 Conclusion

To summarize our key findings, only free bilirubin solutions were found to significantly influence the electrical behavior of our OECT devices. Under low voltage conditions (V_D_ = −0.3 V and V_G_ = 0.3 V), we observed an increasing trend in current ratio with bilirubin concentration when using polarizable gate electrodes (Au, Pt, and GCE), with Au electrodes showing the strongest response (current ratio reaching 8). In contrast, measurements conducted at higher voltages (V_D_ = −0.6 V and V_G_ = 0.8 V for Au, Pt, GCE; V_G_ = 0.4 V for Ag/AgCl) revealed an opposite trend, with current ratios decreasing with concentration (Au electrodes reaching a minimum ratio of 0.6). Importantly, these effects were only observed with polarizable electrodes ‐ the non‐polarizable Ag/AgCl electrode showed negligible current ratio changes under all conditions.

Several factors contribute to these observations. First, the electrical circuit formed during OECT measurements plays a crucial role. Bilirubin's tetrapyrrole structure and carboxylate anion groups can de‐dope the PEDOT:PSS channel, enabling sensitivity.^[^ ^47^ ^]^ As illustrated in Scheme 1b, two capacitive layers form during measurement: one between the gate electrode and electrolyte (C_G_), and another between the electrolyte and channel material (C_CH_). These act as series capacitors, with equivalent capacitance $C_{EQ}=\frac{1}{C_{G}}+\frac{1}{C_{CH}}.$ For non‐polarizable Ag/AgCl electrodes, C_G_ is much larger than C_CH_, causing most voltage drop to occur near the channel region. In contrast, polarizable electrodes (Au, Pt, GCE) have C_G_ and C_CH_ of comparable magnitude.^[^ ^56^, ^57^ ^]^ At V_D_ = −0.3 V and V_G_ = 0.3 V, the increasing current ratio with bilirubin concentration suggests enhanced gating efficiency for polarizable electrodes, likely because C_G_ becomes more dominant as bilirubin concentration increases. The Ag/AgCl electrode shows no such effect because its much larger C_G_ remains unchanged regardless of C_CH_ variations.

At higher voltages (V_D_ = −0.6 V and V_G_ = 0.8 or 0.4 V), additional factors come into play. Bilirubin molecules may undergo structural changes to different isomers, as shown in Scheme 1c. The carbon single or double bonds (indicated by orange arrows) allow rotation of functional groups (dashed square), affecting the formation of intramolecular, intermolecular, or a combination of the two types of hydrogen bonds within bilirubin molecules or with water molecules. The structural change of bilirubin and the formation of hydrogen bonds can influence the electron transfer between the electrolyte and gate/working electrode. Scheme 1d illustrates some possible ways of this formation process. This isomerization and the rearrangement of hydrogen bonds, supported by our CV results showing redox activity at high potentials, likely contributes to the reversed current ratio trend observed at higher voltages.

The HSA‐BR complex behaves fundamentally differently due to hydrogen bonding between bilirubin and HSA amino acids, which restricts bilirubin's structural flexibility.^[^ ^53^ ^]^ These intermolecular hydrogen bonds make the complex more electrochemically stable than free bilirubin, as evidenced by both OECT and CV measurements showing no significant current modulation or redox activity. This stability arises because bilirubin's electron‐donating groups are engaged in binding HSA rather than interacting with the channel material.

In conclusion, PEDOT:PSS‐based OECTs can detect free bilirubin through the molecule's electron‐donating capability, but only when using polarizable gate electrodes (Au, Pt, GCE) where redox reactions and C_G_/C_CH_ relationships enable sensitivity. HSA complexation eliminates this sensitivity by stabilizing bilirubin's structure. Overall, we believe that this work provides crucial insights into how gate materials and measurement conditions affect bilirubin detection in OECTs, explaining why free bilirubin is more readily detected than albumin‐bound bilirubin.

The main drawback of our measurement system is that we need a relatively large OECT setup, and a larger volume of specimens is needed. For future improvement and practical application, we believe that it is essential to decrease the overall size of our setup; for instance, printable OECTs can be designed and constructed, the three terminals can be printed on the same surface, and the size of the electrodes can be decreased. It is also better to have a soft and wearable platform to be used in the medical diagnostic and monitoring process, so the patients can monitor their bilirubin levels in real time. To improve the accuracy of sensitivity, another modification that can be done in the future is to integrate several OECT devices together and conduct the sensing measurement at the same time. By doing so, we are able to have a higher accuracy about the actual sensitivity compared to only one device each time.

For future clinical applications in neonatal jaundice monitoring, several approaches could overcome the challenge of detecting protein‐bound bilirubin in serum. Enzymatic methods using bilirubin oxidase or albumin‐specific proteases could liberate free bilirubin for detection. Alternatively, techniques to temporarily disrupt the HSA‐BR complex without denaturing the protein could be developed. Another possibility involves engineering the sensor interface to facilitate electron transfer directly from the protein complex. These advancements could lead to practical OECT‐based bilirubin sensors for point‐of‐care neonatal care, potentially replacing current invasive blood tests with non‐invasive or continuous monitoring solutions. The fundamental understanding gained here of bilirubin‐electrode‐channel interactions provides a solid foundation for these future developments in electrochemical bilirubin sensing technologies.

## 4 Experimental Section

### OECT Device Fabrication

The substrates used in the OECT fabrication process were ITO‐coated glass (20 mm × 20 mm × 0.7 mm glass with 4 mm × 20 mm ITO stripes). The substrates were completely cleaned before the spin‐coating process. First, the substrates were immersed in soap water and ultrasonicated for 15 min, then thoroughly washed with DI water. After this, the substrates were air‐dried and immersed in isopropanol, then ultrasonicated for another 15 min. Following this step, the substrates were air‐dried again, then immersed in acetone and ultrasonicated for 15 min. After final air‐drying, the substrates were ready for use.

The PEDOT:PSS solution (Clevios PH 1000) was prepared using the following procedure: 10 mL of pristine PEDOT:PSS solution was filtered through a 0.22 µm syringe filter. To increase its conductivity, 0.5 mL of ethylene glycol, 0.1 mL of (3‐glycidyloxypropyl) trimethoxysilane (GOTMS), and ≈20 µL of dodecylbenzenesulfonic acid (DBSA) (all from Sigma) were added to the 9.5 mL of filtered PEDOT:PSS solution.^[^ ^58^, ^59^ ^]^ The mixture was gently vortexed for 10 s and then ultrasonicated for 10 min at room temperature. Throughout the entire process, both the pristine PEDOT:PSS solution and the mixture were covered with aluminum foil to avoid light exposure. The mixture was stored at 4 °C for future use, with a maximum shelf life of one week. The mixture was filtered again immediately before the spin‐coating process.

The PEDOT:PSS mixture was spin‐coated onto the clean ITO‐coated substrates at 2000 rpm for 60 s. After spin‐coating, the substrates were heated in an oven at 100 °C for 1 h without light exposure. Subsequently, a glass cylinder with a diameter of 1 cm was attached to the PEDOT:PSS surface using PDMS. The glass cylinders were cleaned following the same process as the ITO‐coated substrates. The mass ratio of PDMS to its curing agent was maintained at 10:1. After attaching the glass cylinders, the devices were ready for testing.

### Free Bilirubin and HSA‐BR Complex Solution Preparation

For free bilirubin solution preparation, bilirubin powder was added to 0.1 M KCl solution, then 2 M NaOH was added dropwise to the suspension until complete dissolution was achieved. The solution was gently vortexed and then sonicated for 5 min. All steps were performed in the dark. Different concentrations of free bilirubin solution were obtained through sequential dilution. For the HSA‐BR complex solution, bilirubin was first dissolved in 0.1 M KCl using the same procedure. HSA solution was prepared by dissolving HSA in 0.1 M KCl solution. The HSA solution was then mixed with the free bilirubin solution.

### Electrochemical and Absorption Spectroscopy Characterization

The polarizable disk electrodes were cleaned before any measurements. For Au and Pt electrodes, they were polished on a cloth pad using 0.1 and 0.03 µm alumina slurry for 5 min each, then thoroughly washed with water. After polishing, they were immersed in 0.5 M H_2_SO_4_, and cyclic voltammetry measurements were performed from −0.2 to 1.5 V for ten cycles. Following this, they were washed with DI water, then sonicated in absolute ethanol and DI water for 5 min each. For the GCE electrode, the process was identical except for the H_2_SO_4_ cleaning step.

The OECT measurements were performed using a Keithley 2612B source meter with a probe station. The measurement conditions were either V_D_ = −0.3 V with V_G_ = 0.3 V or V_D_ = −0.6 V with V_G_ = 0.8 V (0.4 V for Ag/AgCl). CV measurements were conducted using the same OECT device architecture but with a standard three‐electrode setup: Ag/AgCl as the reference electrode, Pt wire as the counter electrode, and Au, Pt, or glassy carbon as the working electrode. The measurements were performed using a Gamry Reference 620 potentiostat, with a potential range of −0.5 to 0.9 V and a scan rate of 10 mV s^−1^. UV–vis measurements were conducted using a SpectraMax iD3 Multi‐Mode Microplate Reader to obtain absorption spectra. SEM images are taken before and after OECT measurements using a Gemini Ultra 55 field emission scanning electron microscope (Zeiss) at 3 kV with an SE2 detector, which were indicated in Figure S14 (Supporting Information) to prove the robustness of OECT devices. Images were analyzed using ImageJ. Details of selectivity experiments are illustrated in the Supporting Information.

### Statistical Analysis

All data were presented as the means ± standard deviation (SD) of at least three samples unless otherwise reported.

## Conflict of Interest

The authors declare no conflict of interest.

## Supporting information



Supporting Information

## Data Availability

The data that support the findings of this study are available from the corresponding author upon reasonable request.
