# Supplementary material for: Bilirubin Sensing Using Organic Electrochemical Transistors: Role of Gate Materials and Operational Parameters
Source: Adv Healthc Mater. 2025 Aug 7;14(31):e02481. doi: 10.1002/adhm.202502481 (PMC12683225; doi:10.1002/adhm.202502481)
Supplement: Supplementary file 1 — Supporting Information [file ADHM-14-0-s001.docx]

Supporting Information

**Bilirubin Sensing Using Organic Electrochemical Transistors: Role of Gate Materials and Operational Parameters**

*Yunjia Song, Sihui Xu, Onur Parlak**


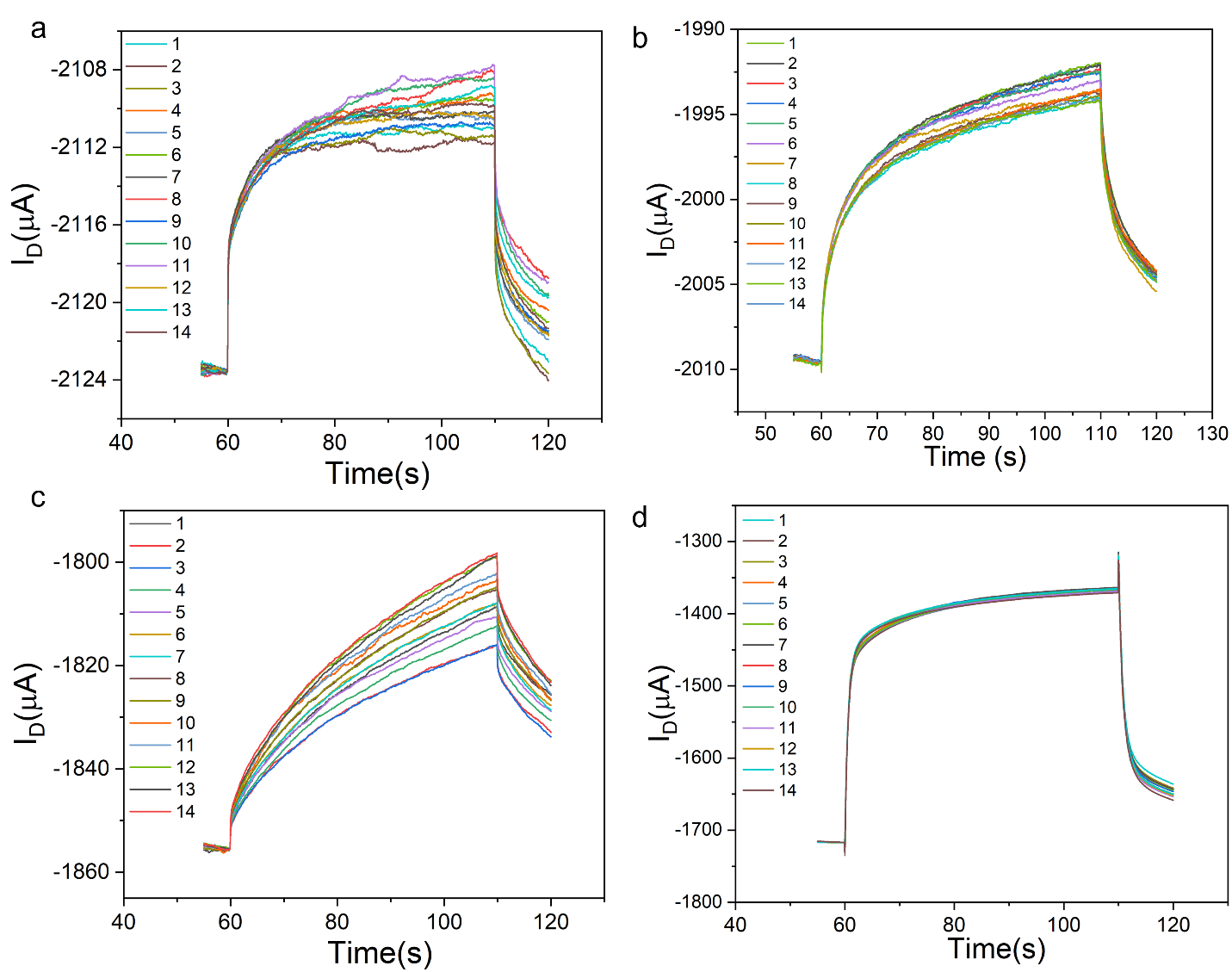
**Figure S1**. Control experiments with (a) Au (b) Pt (c) glassy carbon (d) Ag/AgCl gate electrode. Measurements with all electrodes were done under V_D_=-0.3 V, V_G_=0.3 V with 0.1M KCl solution (with a small amount of NaOH to reach pH 7.7).

**
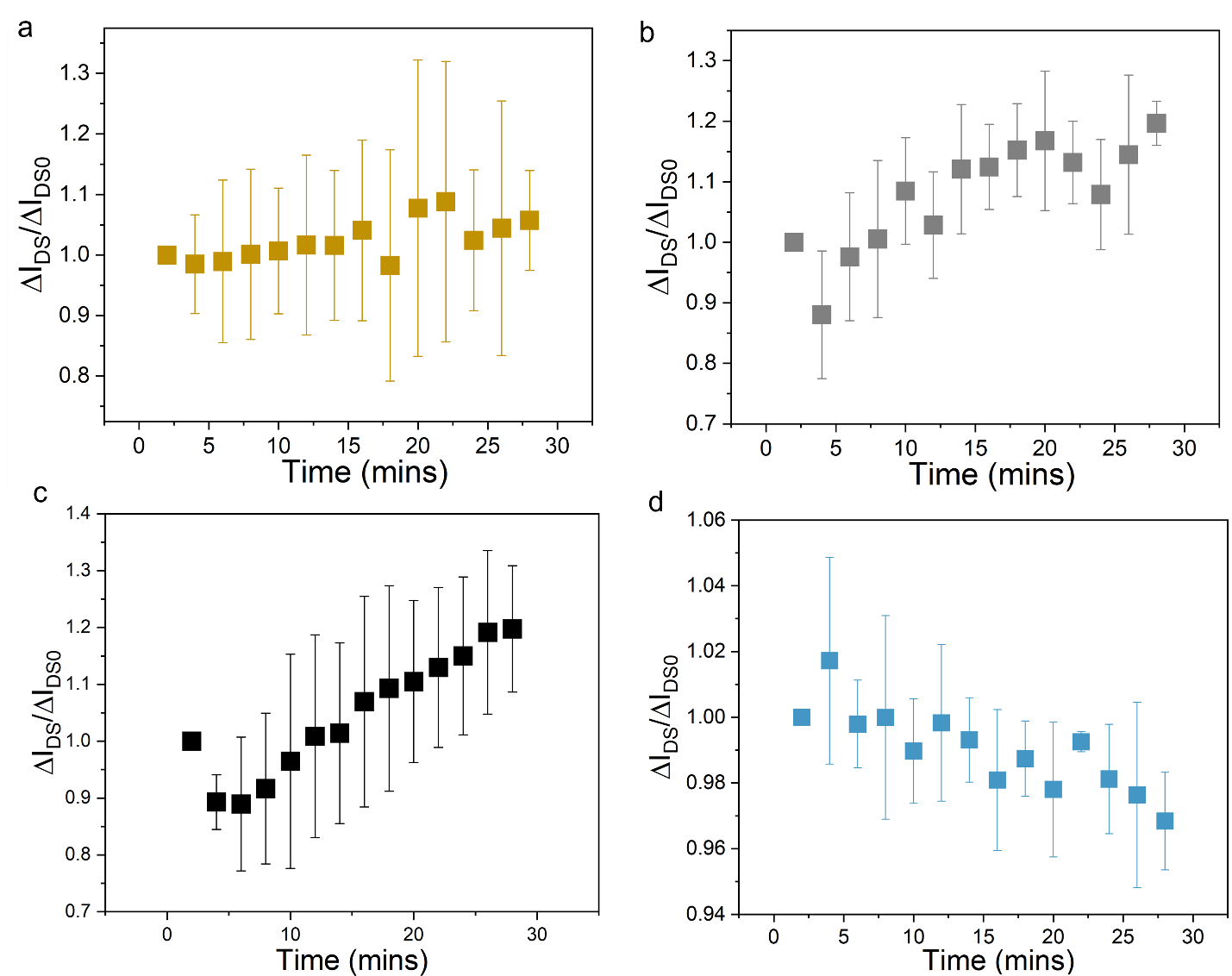
Figure S2.** Drain-source current ratio changes of control experiments vs time with (a) Au (b) Pt (c) glassy carbon (d) Ag/AgCl gate electrode. Measurements with all electrodes were done under V_D_=-0.3 V, V_G_=0.3 V with 0.1M KCl solution (with a small amount of NaOH to reach pH 7.7).


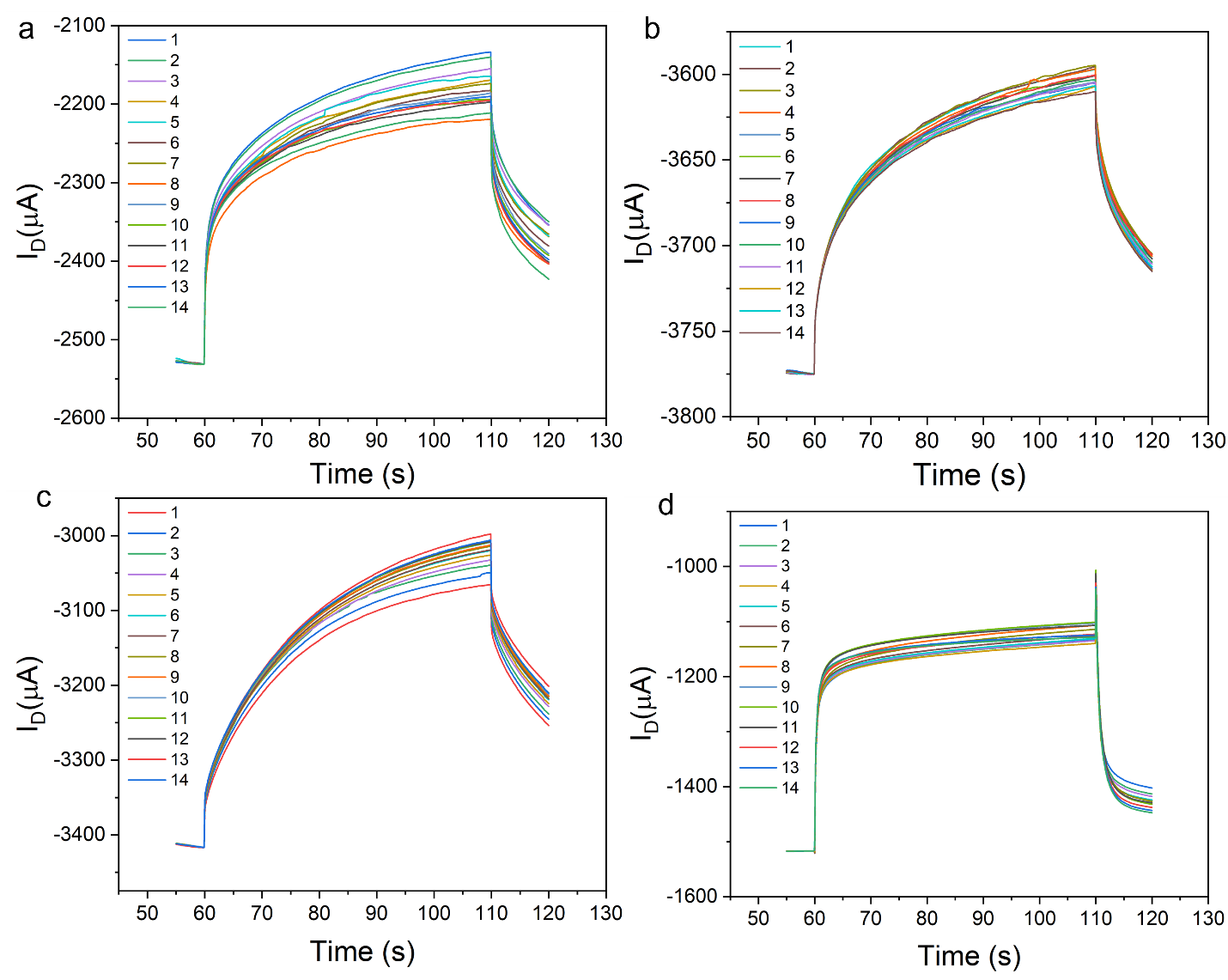
**Figure S3**. Control experiments with (a) Au (b) Pt (c) glassy carbon (d) Ag/AgCl gate electrode. Measurements with Au, Pt, and GCE electrodes were done under V_D_=-0.6 V, V_G_=0.8 V, and measurements with Ag/AgCl electrode were done under V_D_=-0.6 V, V_G_=0.4 V with 0.1M KCl (with a small amount of NaOH to reach pH 7.7).


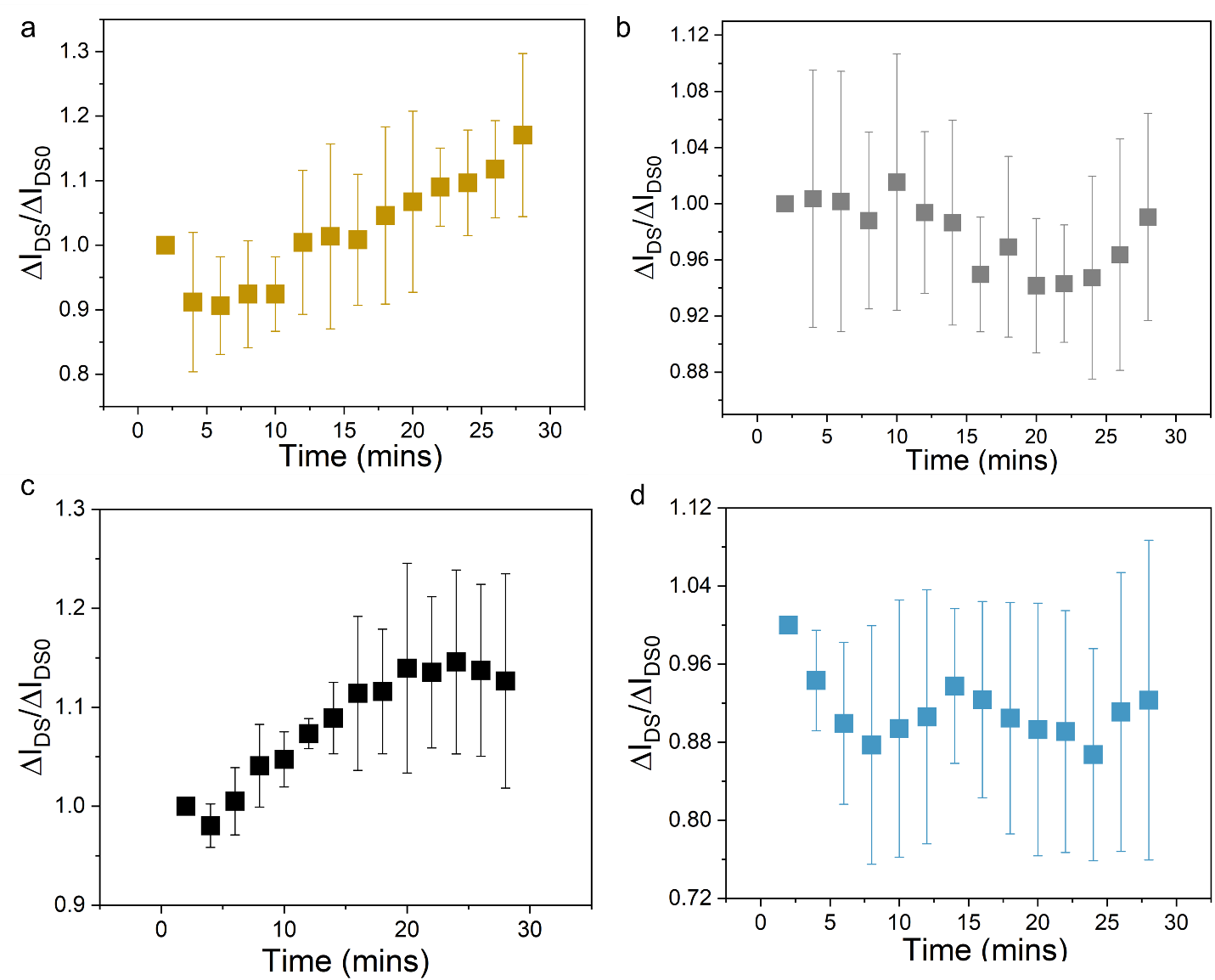


**Figure S4**. Drain-source current ratio changes of control experiments vs time with (a) Au (b) Pt (c) glassy carbon (d) Ag/AgCl gate electrode. Measurements with Au, Pt, and GCE electrodes were done under V_D_=-0.6 V V_G_=0.8 V, measurements with Ag/AgCl were done under V_D_=-0.6 V V_G_=0.4 V with 0.1M KCl solution (with a small amount of NaOH to reach pH 7.7).

**
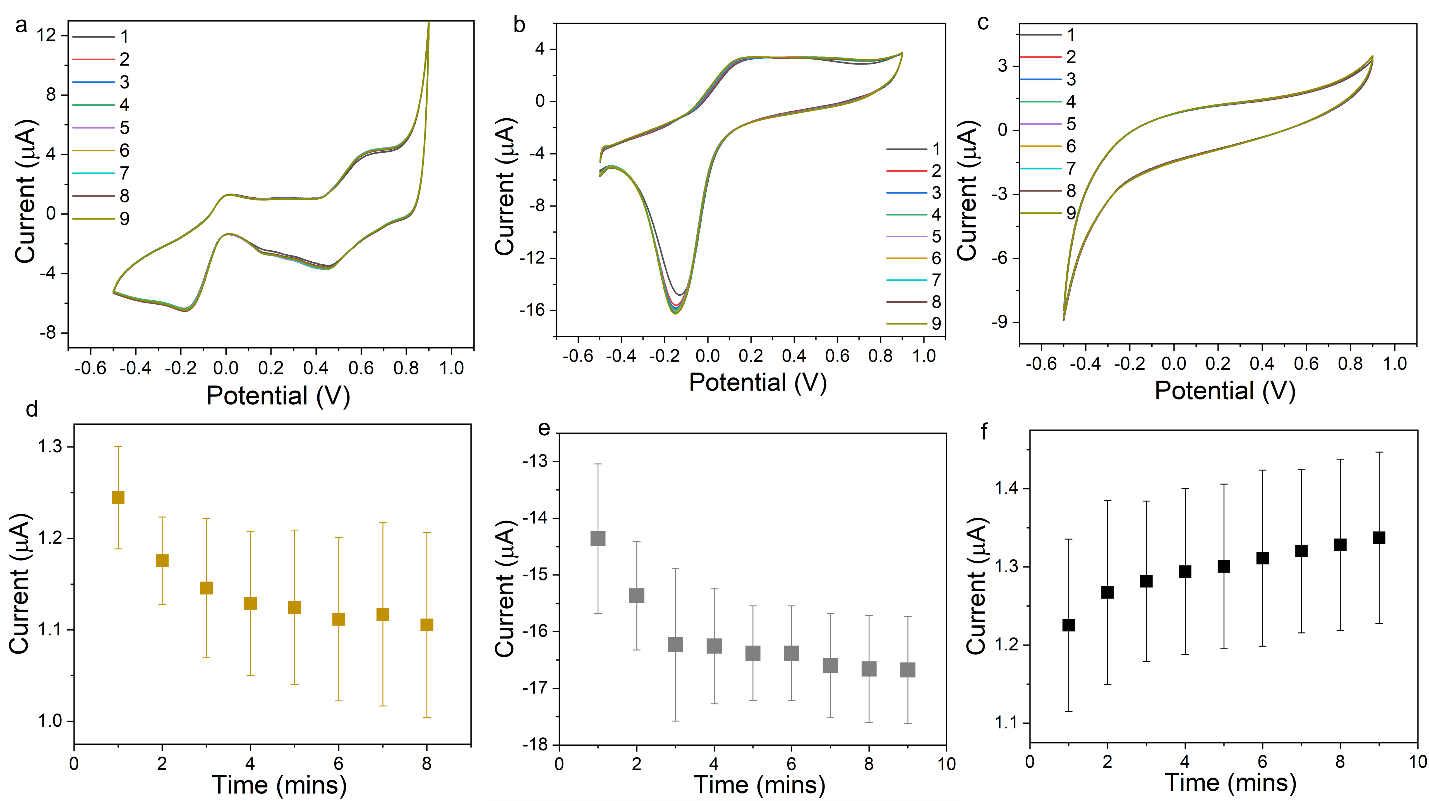
**

**Figure S5**. Control experiments of CV measurements (a) Au (b) Pt (c) glassy carbon working electrode. Peak current changes vs time with (d) Au (e) Pt (f) glassy carbon working electrode. All measurements were done in 0.1M KCl solution (with a small amount of NaOH to reach pH 7.7).

**
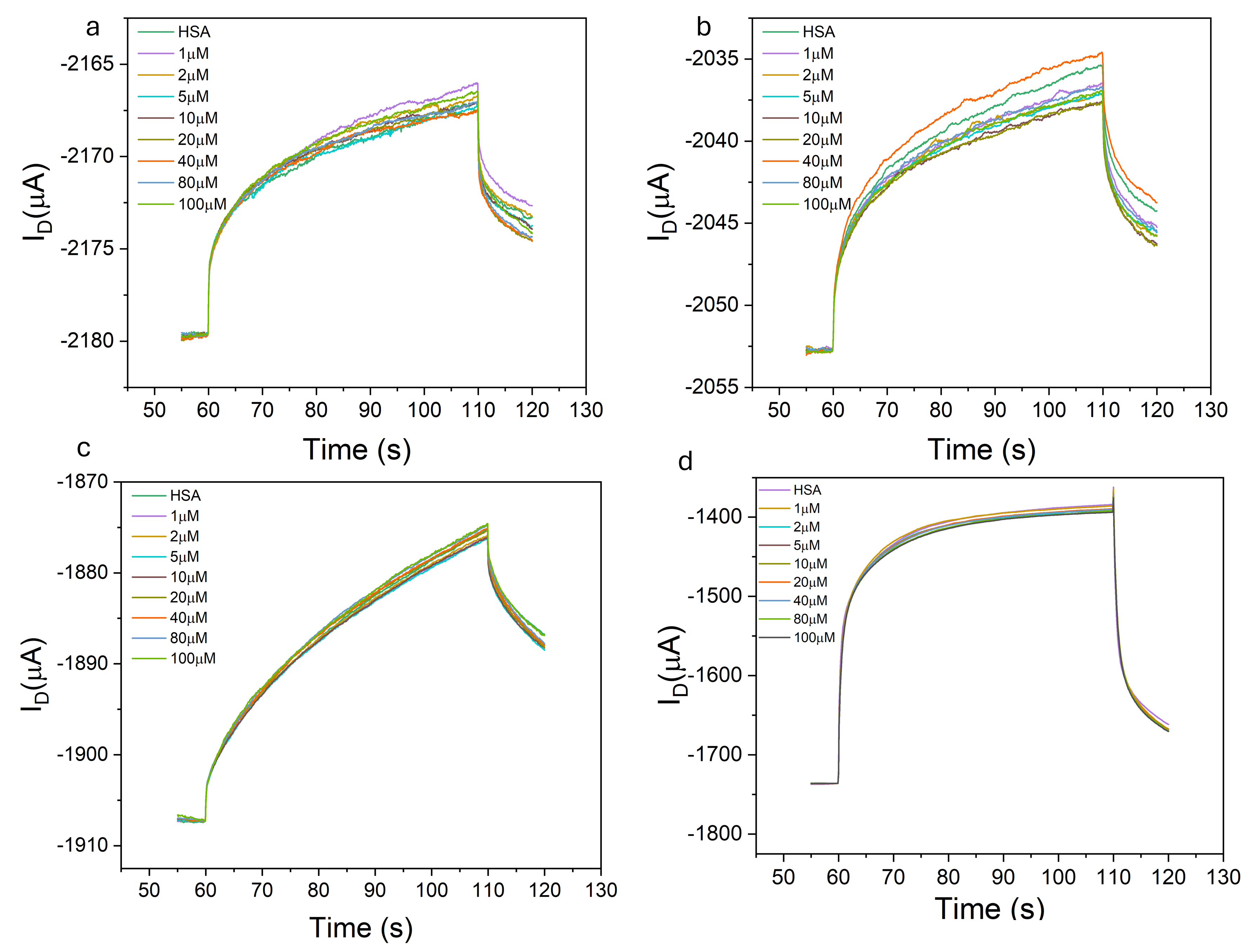
**

**Figure S6**. Drain-source current changes of main experiments vs time with (a) Au (b) Pt (c) glassy carbon (d) Ag/AgCl gate electrode. All measurements were done under V_D_=-0.3 V, V_G_=0.3 V in different concentrations of HSA-BR complex solution.


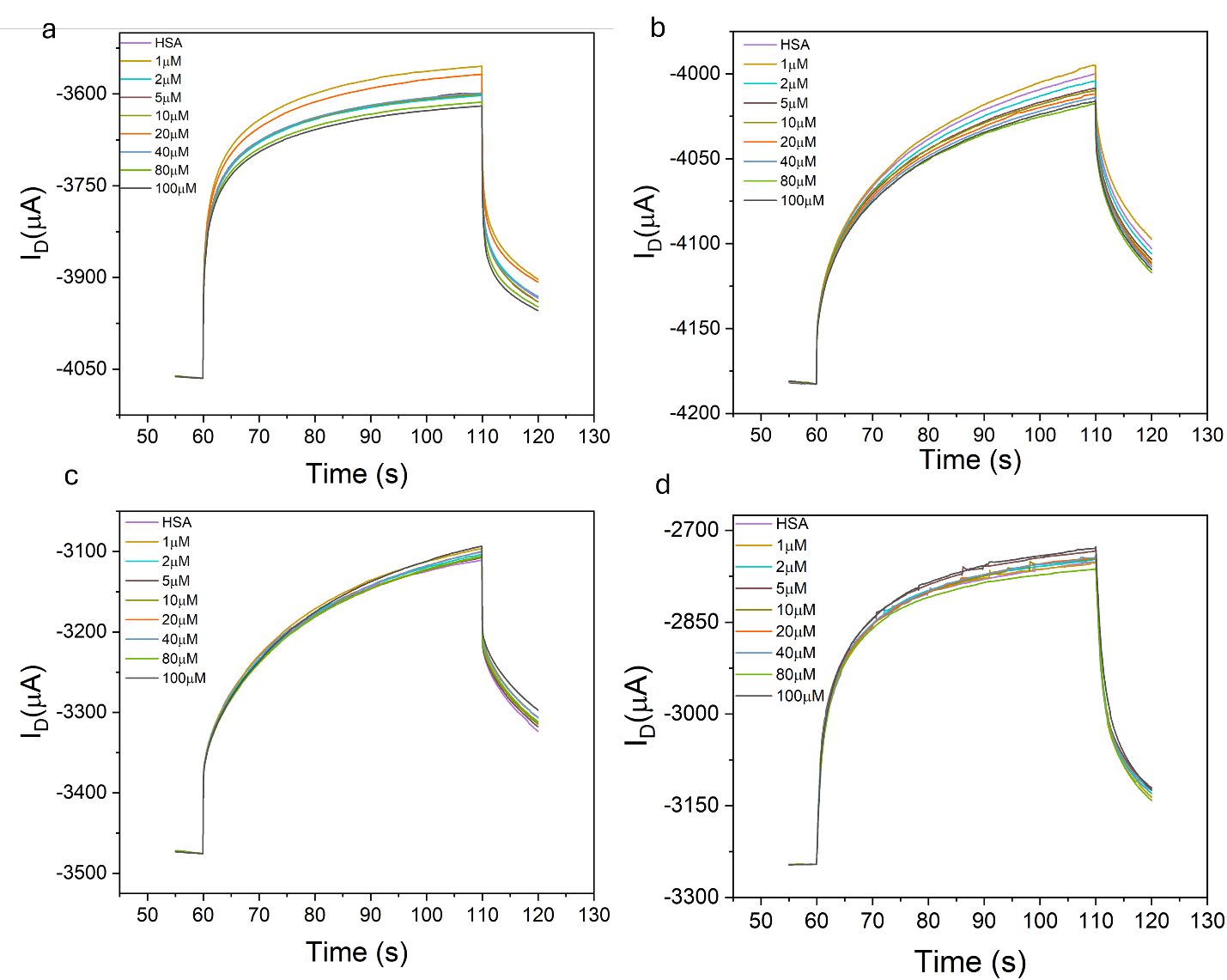
**Figure S7**. Drain-source current changes of main experiments vs time with (a) Au (b) Pt (c) glassy carbon (d) Ag/AgCl gate electrode. Measurements with Au, Pt, and GCE electrodes were done under V_D_=-0.6 V, V_G_=0.8 V, and measurements with Ag/AgCl electrode were done under V_D_=-0.6 V, V_G_=0.4 V. The measurements were done in different concentrations of HSA-BR complex solution.

**
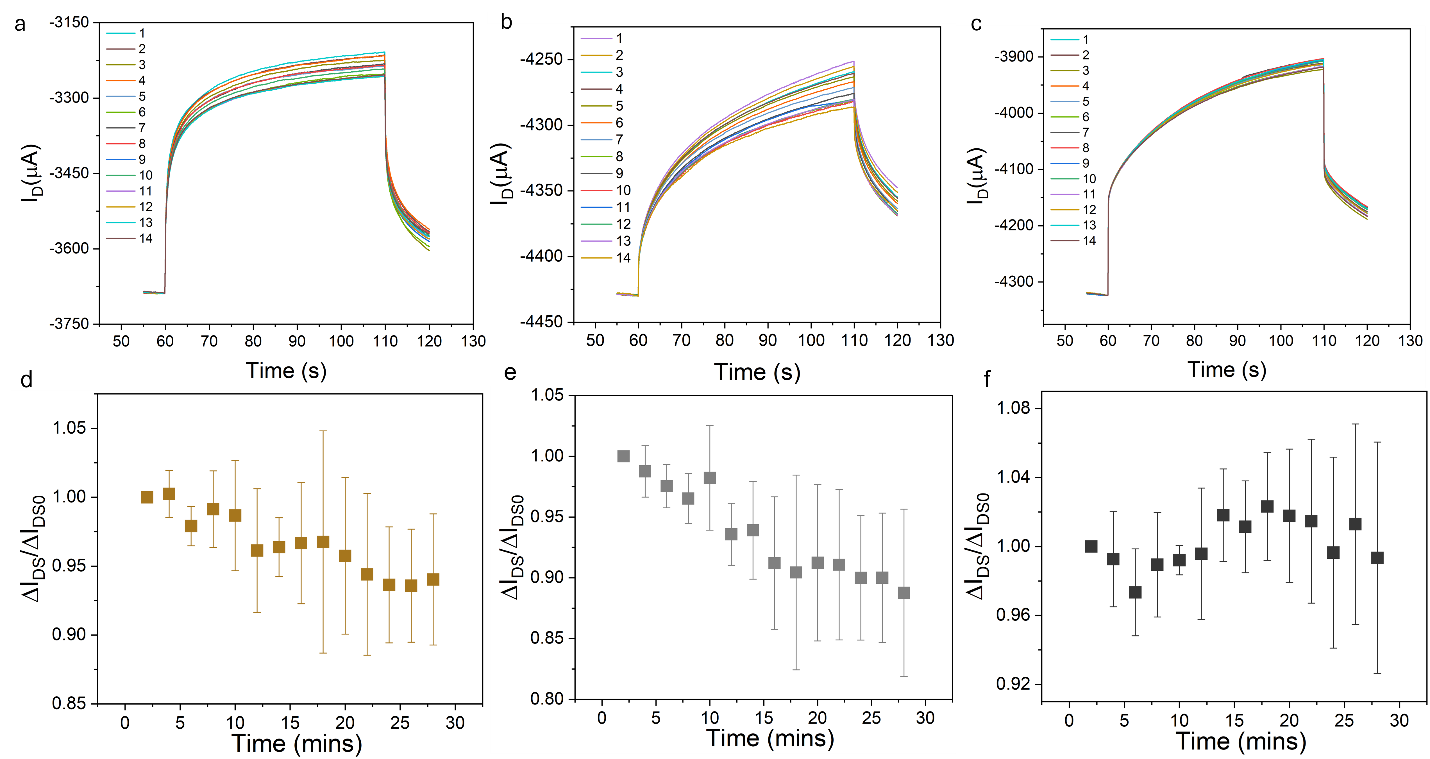
Figure S8**. Control experiments with (a) Au (b) Pt (c) glassy carbon gate electrode. Drain-source current ratio changes vs time of control experiments with (d) Au (e) Pt (f) glassy carbon gate electrode. Measurements with Au, Pt, and GCE electrodes were done under V_D_=-0.6V V_G_=0.8V, in 100µM HSA solution.


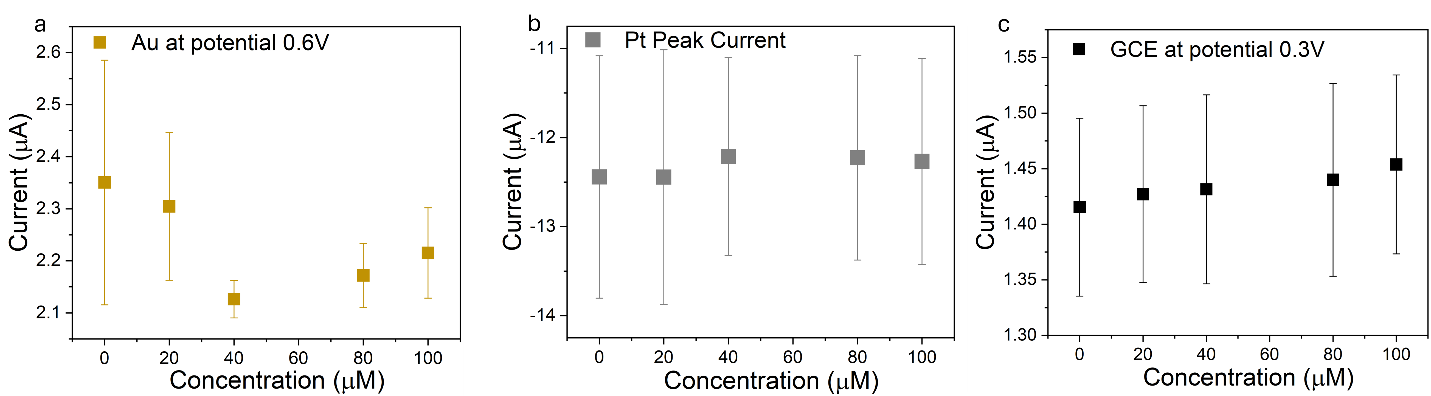
**Figure S9**. CV measurements with (a) Au (b) Pt (c) glassy carbon working electrode. All the measurements were done in different concentrations of the HSA-BR complex solution.


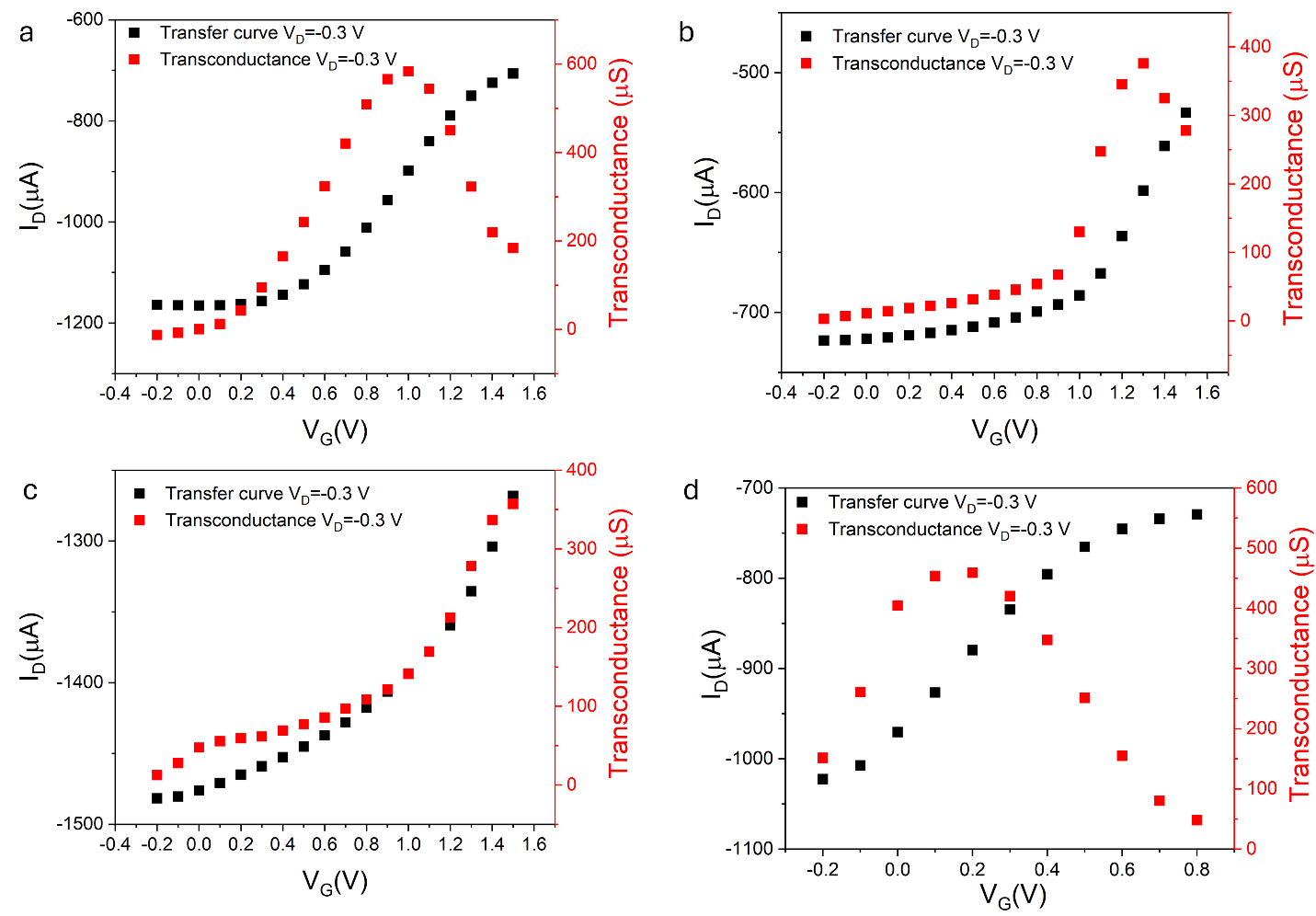
**Figure S10**. Transfer curve and transconductance of (a) Au (b) Pt (c) glassy carbon (d) Ag/AgCl electrode at V_D_=-0.3 V in 0.1M KCl solution (with a small amount of NaOH to reach pH 7.7).


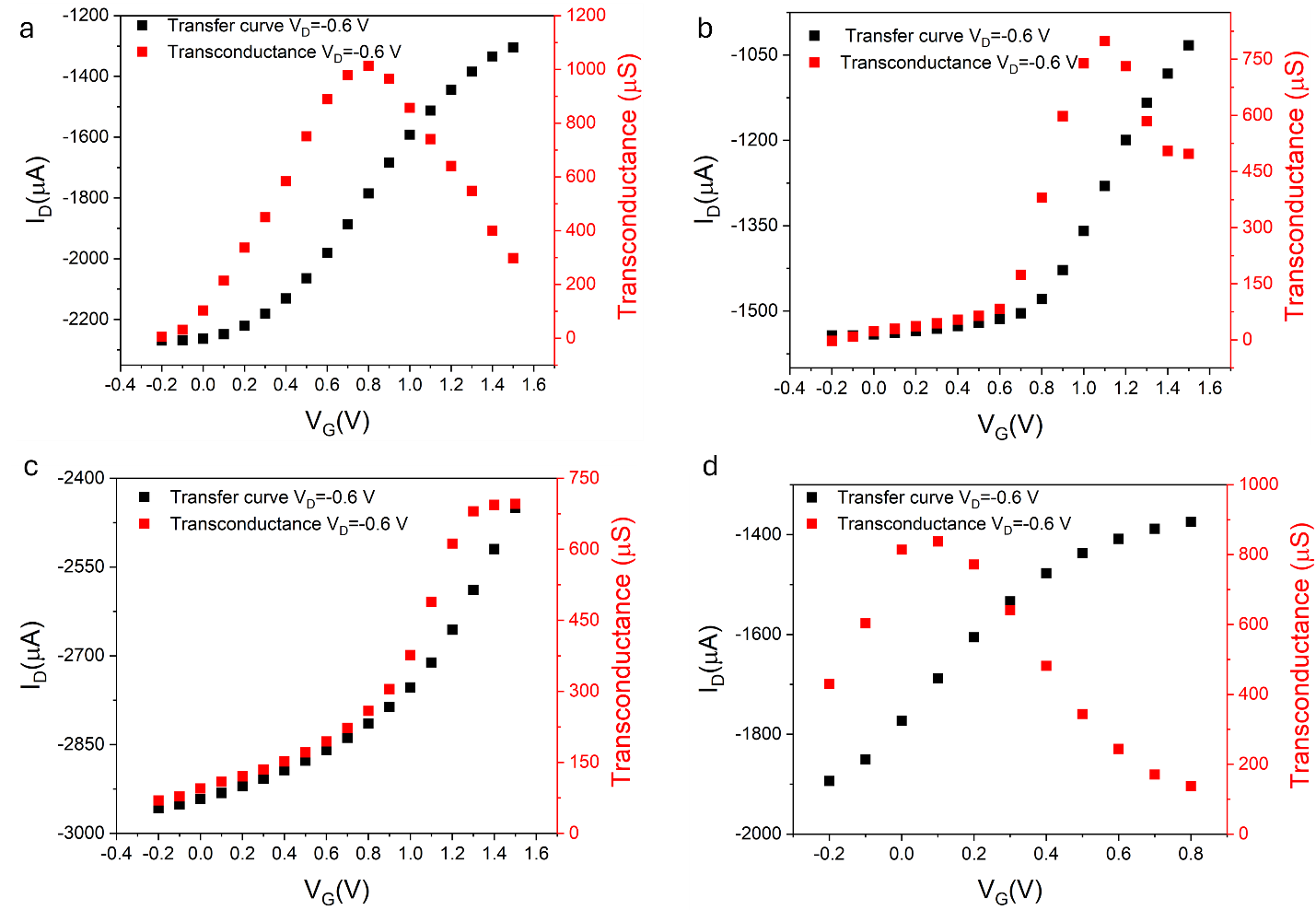


**Figure S11**. Transfer curve and transconductance of (a) Au (b) Pt (c) glassy carbon (d) Ag/AgCl electrode at V_D_=-0.6 V in 0.1M KCl solution (with a small amount of NaOH to reach pH 7.7).


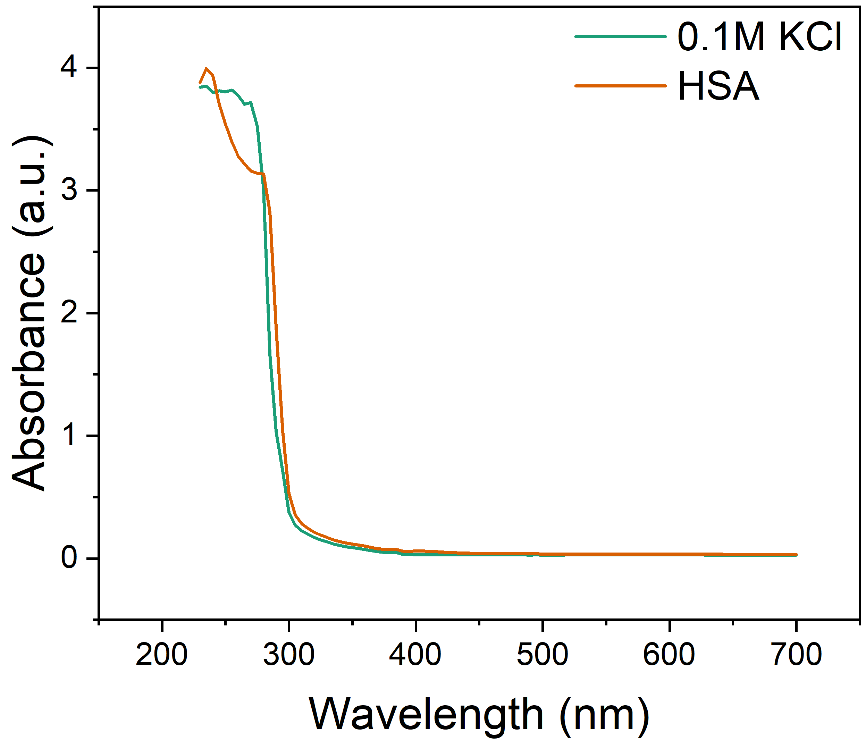


**Figure S12**. UV-vis Spectrum of 0.1M KCl solution and 100µM HSA solution.


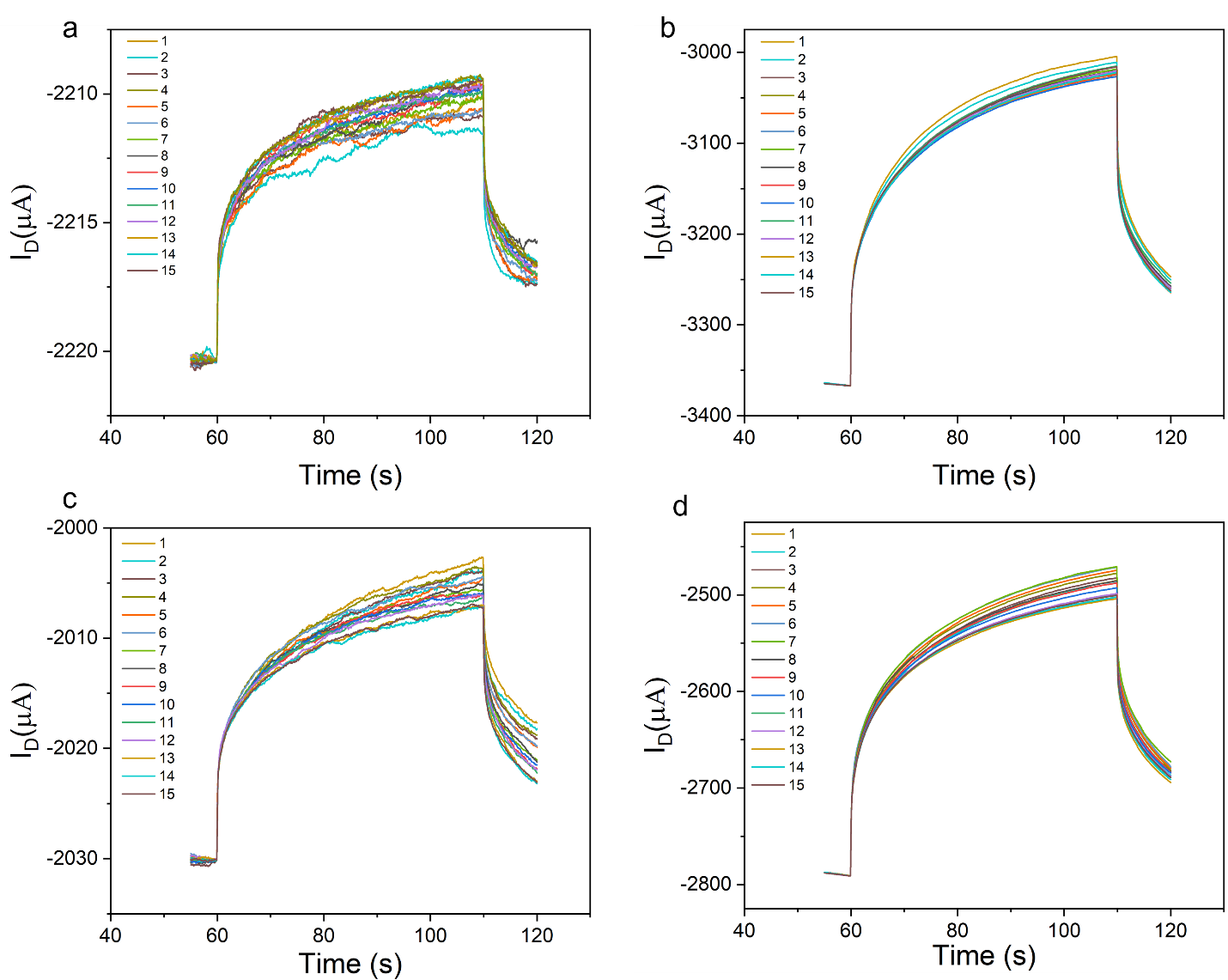


**Figure S13**. Continuous measurement of free bilirubin solution (20 µM) for 30 minutes at (a) V_G_=0.3 V (b) V_G_=0.8 V. Continuous measurement of free bilirubin solution (100 µM) for 30 minutes at (c) V_G_=0.3 V (d) V_G_=0.8 V.


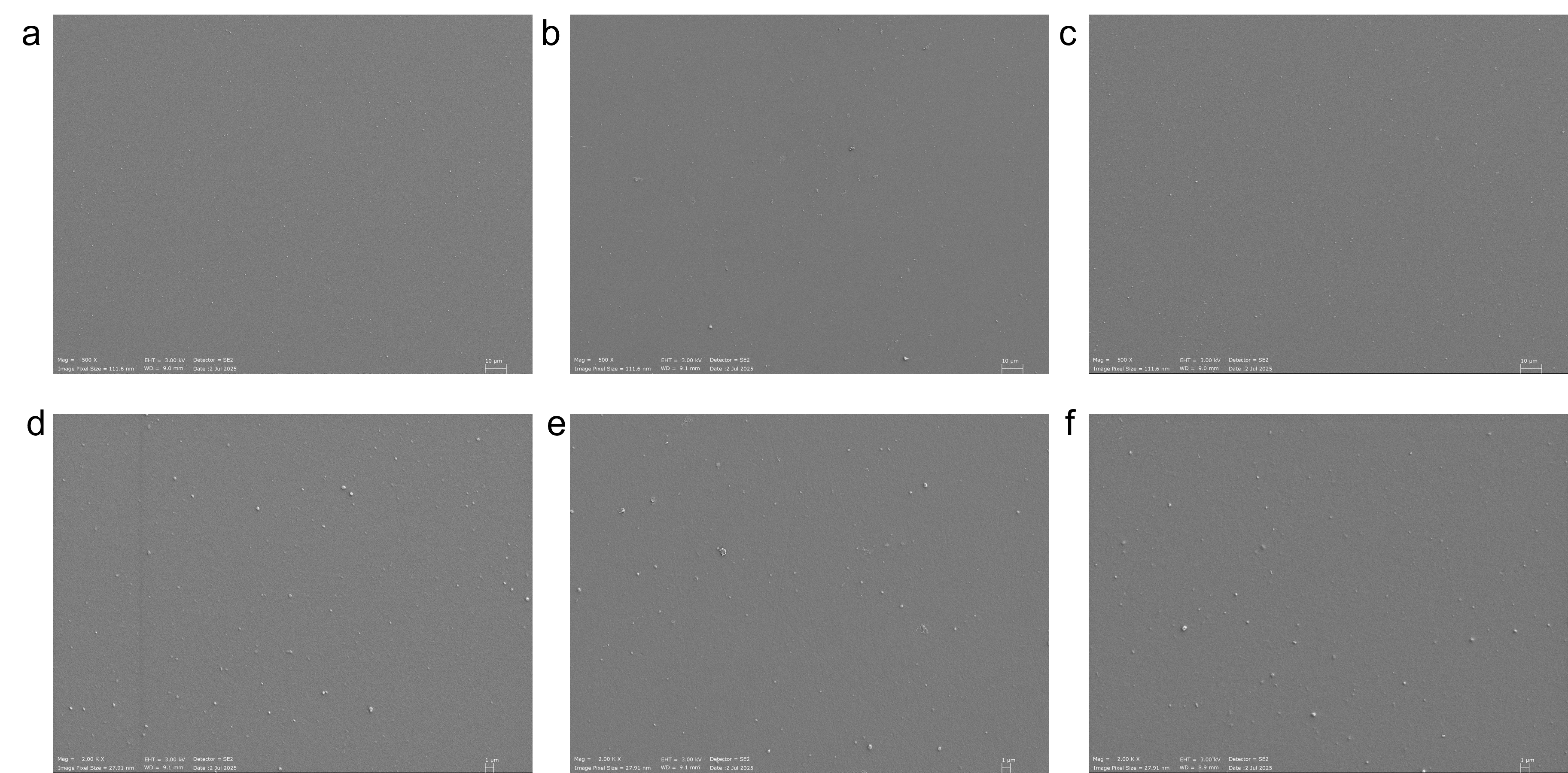


**Figure S14.** (a) Before the OECT measurements at 500X magnification. After the OECT measurements under (b) 0.3 V (c) 0.8 V gate voltage at 500X magnification. (d) Before the OECT measurements at 2000X magnification. After the OECT measurements under (e) 0.3 V (f) 0.8 V gate voltage at 2000X magnification.


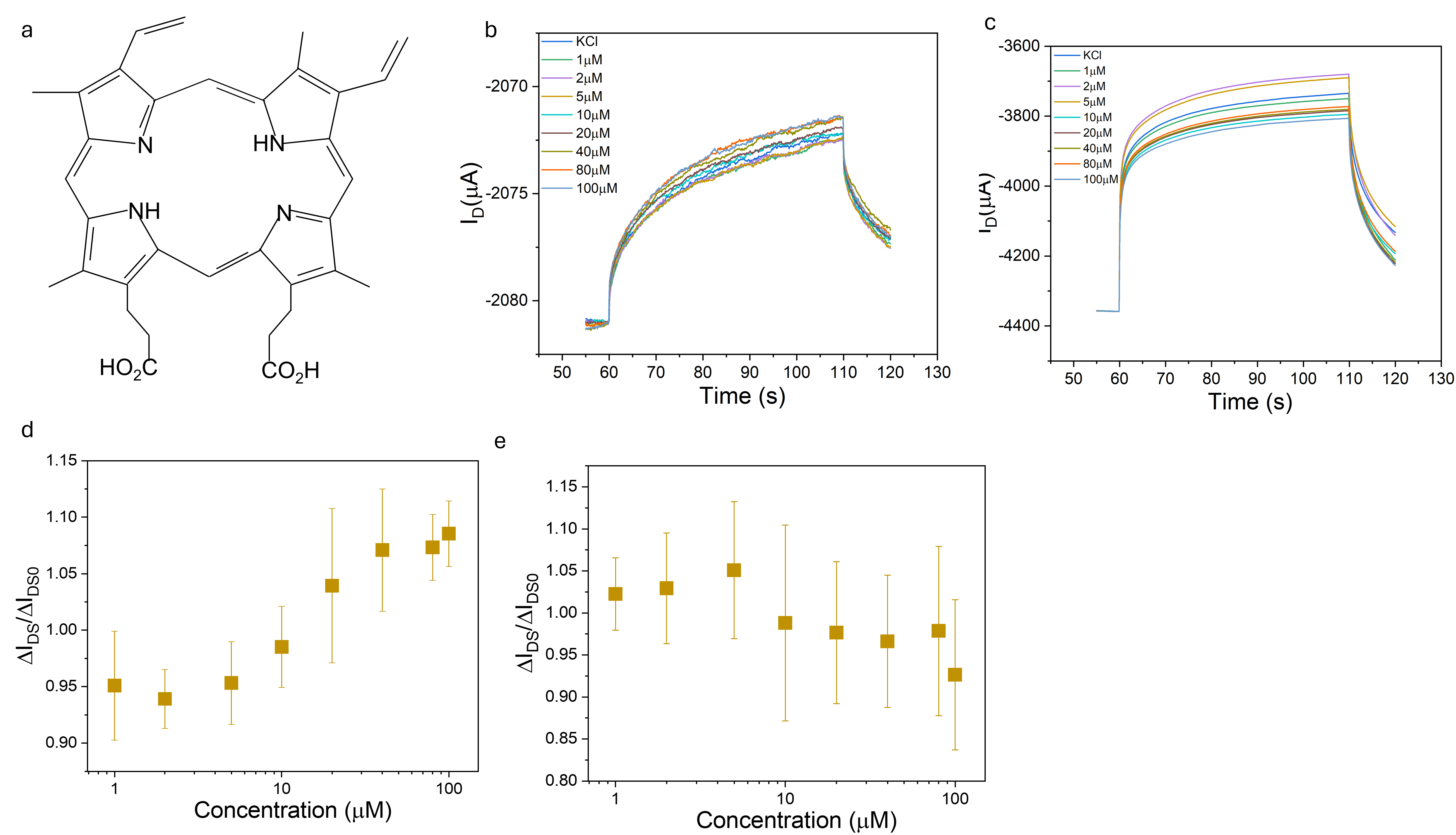


**Figure S15.** (a) Structure of protoporphyrin IX. Drain-source current change vs time with Au gate electrode at (b) 0.3 V (c) 0.8 V gate voltage. Drain-source current ratio changes vs different protoporphyrin IX concentration with Au gate electrode at (d) 0.3 V (e) 0.8 V gate voltage.

Protoporphyrin IX solution was prepared following the same protocol as that used for the free bilirubin solution to prove the selectivity of the free bilirubin sensing.


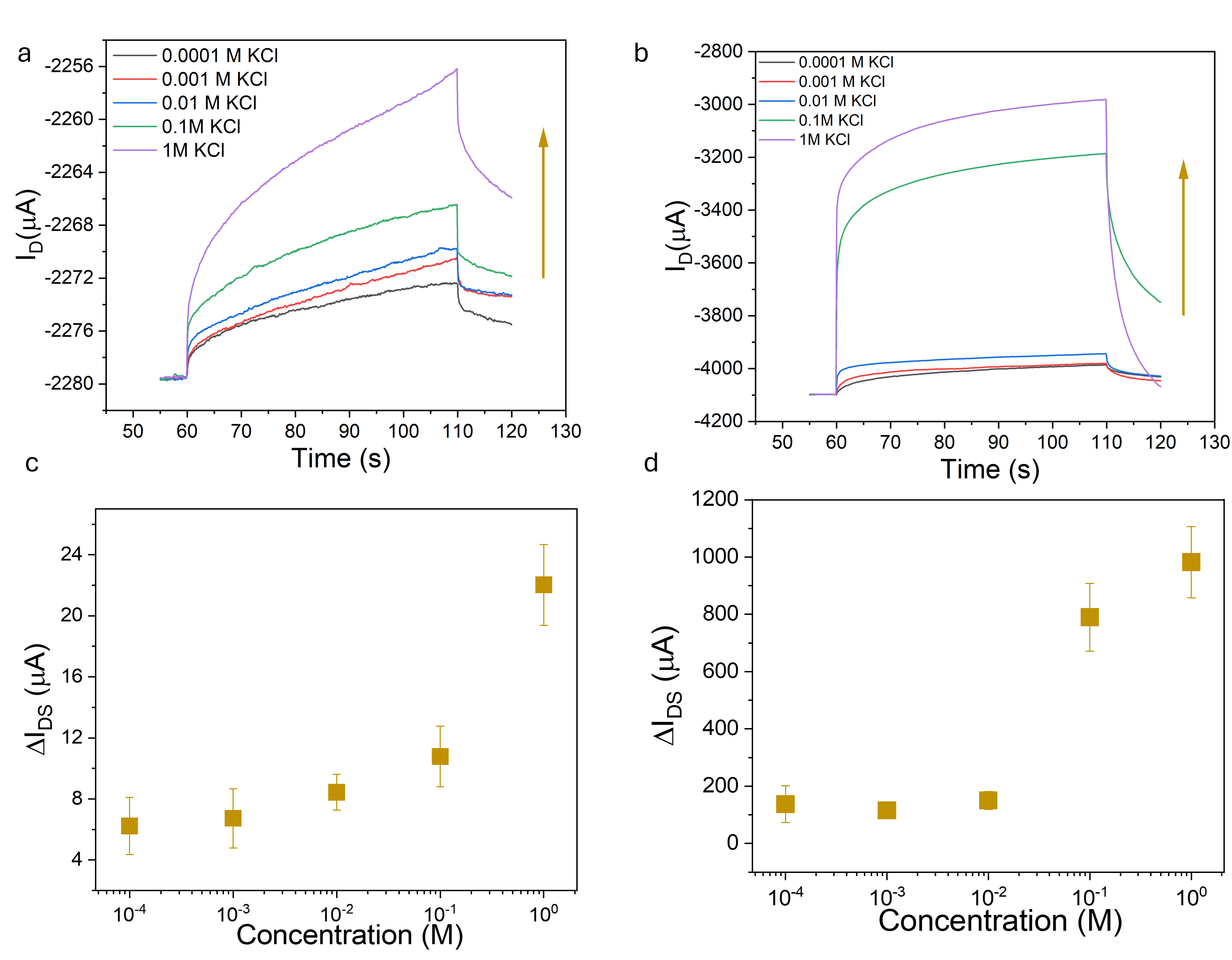


**Figure S16.** Drain-source current change vs time with Au gate electrode at (a) 0.3 V (b) 0.8 V gate voltage. Drain-source current changes vs different KCl solution concentration with Au gate electrode at (c) 0.3 V (d) 0.8 V gate voltage.


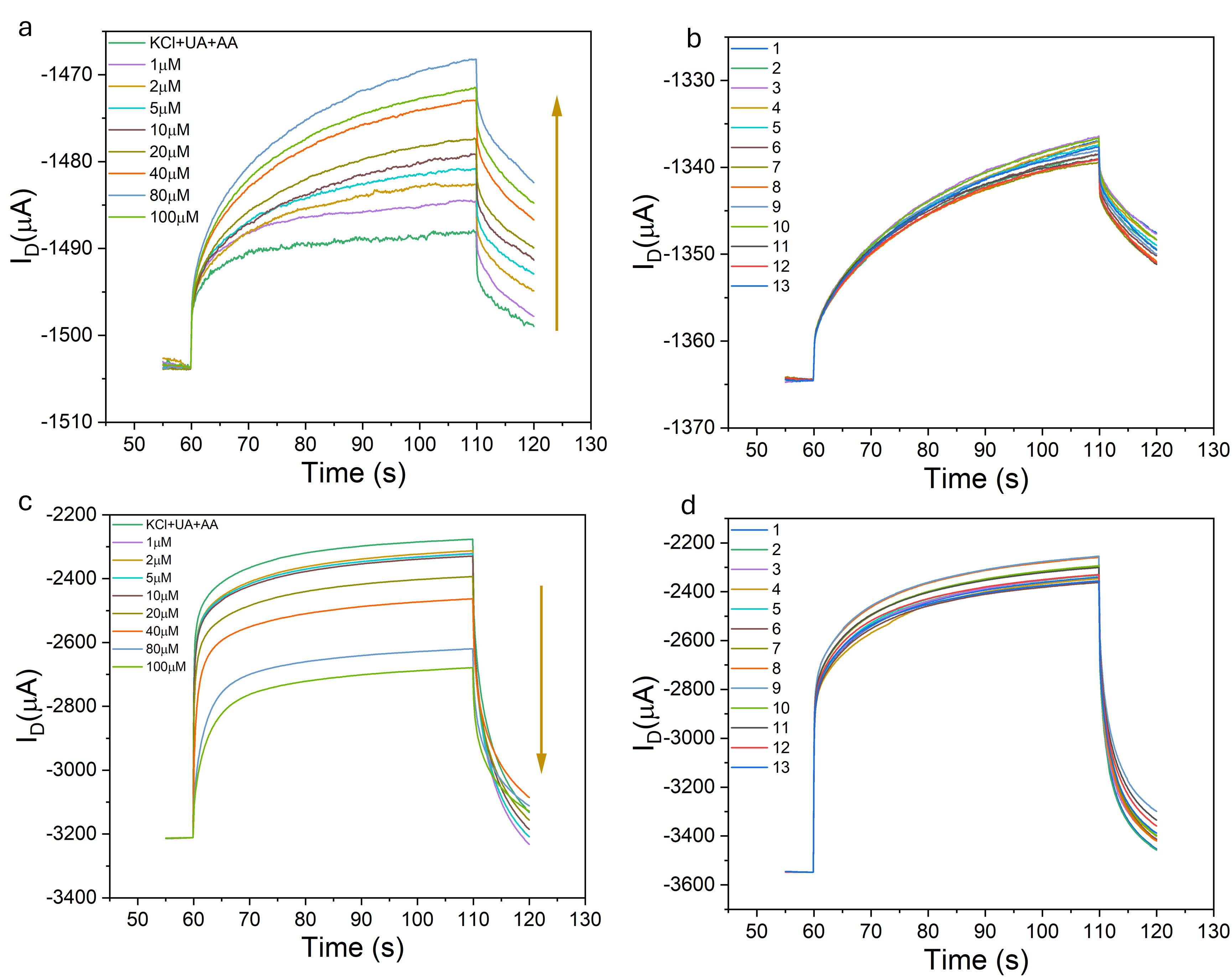


**Figure S17** Drain-source current change vs time with Au gate electrode with different concentrations of bilirubin under (a) 0.3 V (b) 0.8 V gate voltage. Drain-source current change vs time with Au gate electrode with only UA,AA and KCl mixed solution under (c) 0.3 V (d) 0.8 V gate voltage. The arrows indicate the direction of current change vs different bilirubin concentrations (blank-1µM-2µM-5µM-10µM-20µM-40µM-80µM-100µM).

The sensitivity test of free bilirubin was conducted in the presence of 5µM urea (UA) and 100µM ascorbic acid (AA) to prove the possible sensing ability in the physiological condition.


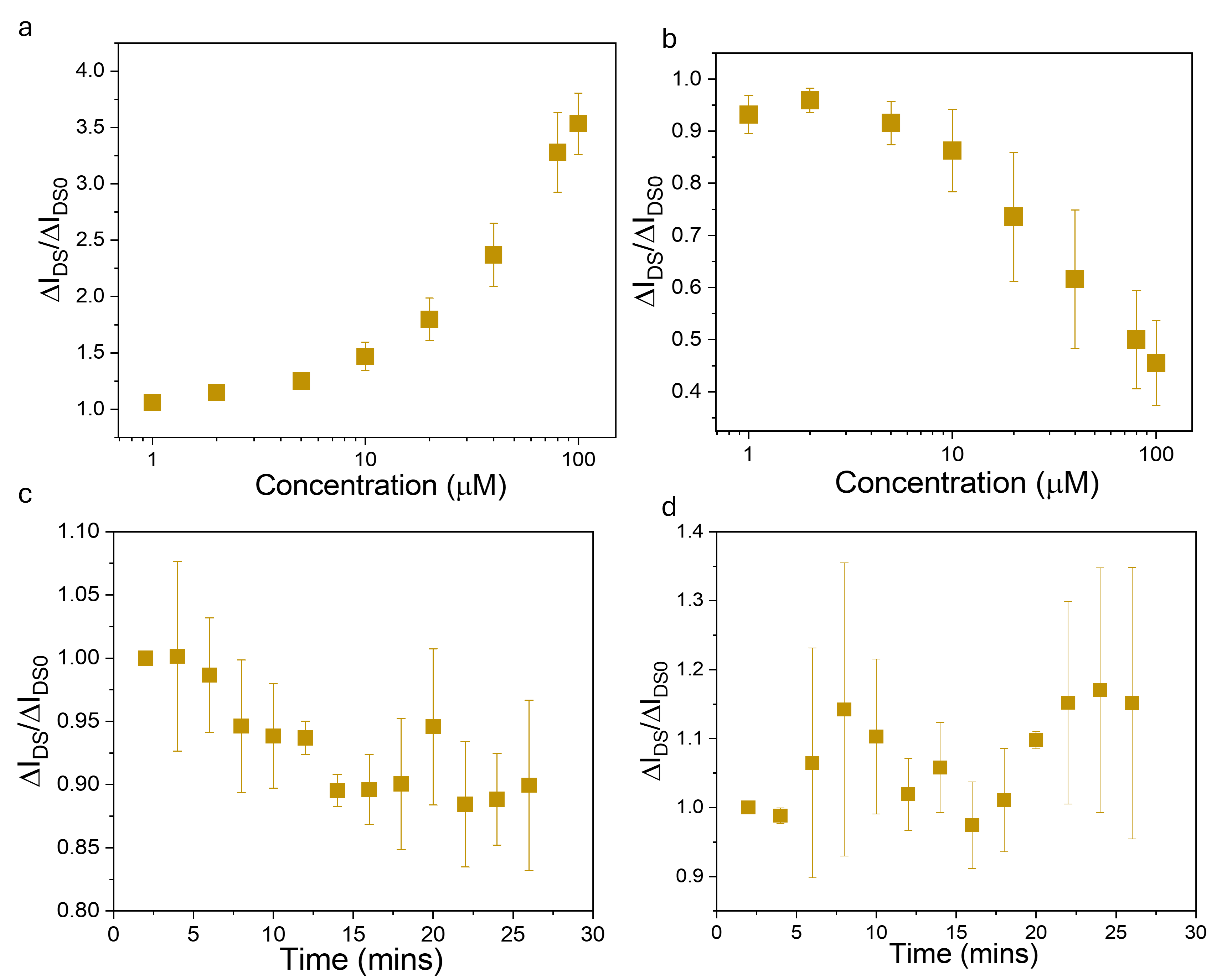


**Figure S18.** Drain-source current ratio changes vs different bilirubin concentration with Au electrode under (a) 0.3 V (b) 0.8 V gate voltage. Drain-source current ratio changes vs time with Au electrode under (c) 0.3 V (d) 0.8 V gate voltage. The blank solution for all measurements is UA, AA, and KCl mix solution.


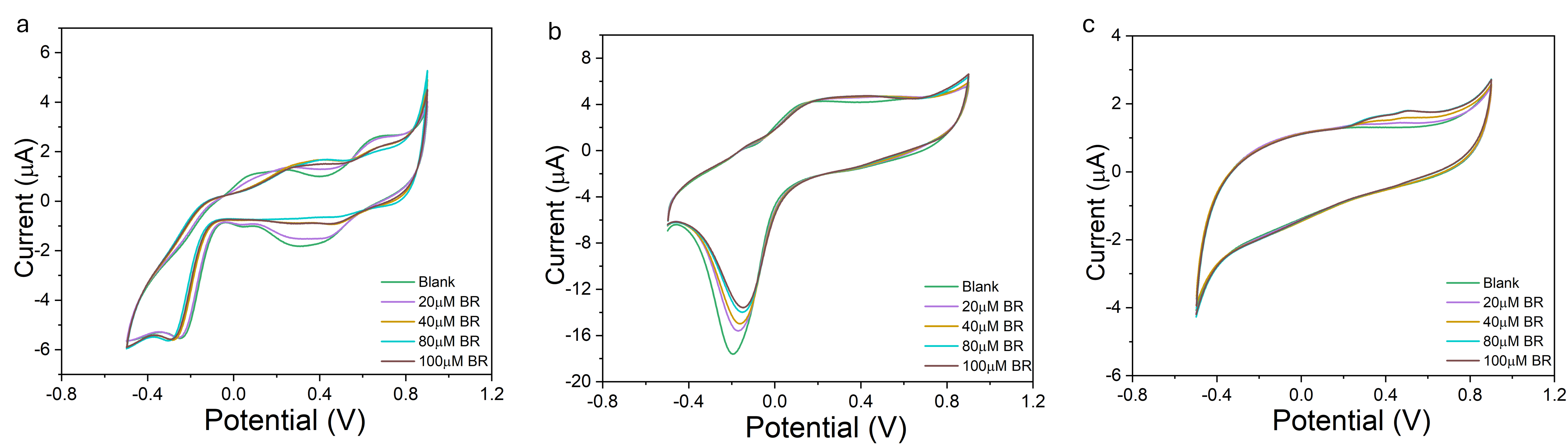


**Figure S19.** CV curves measured with different free bilirubin concentrations with (a) Au (b) Pt (c) GC working electrodes.
